# Major histocompatibility complex class II DR and DQ evolution and variation in wild capuchin monkey species (Cebinae)

**DOI:** 10.1371/journal.pone.0254604

**Published:** 2021-08-12

**Authors:** Janet C. Buckner, Katharine M. Jack, Amanda D. Melin, Valérie A. M. Schoof, Gustavo A. Gutiérrez-Espeleta, Marcela G. M. Lima, Jessica W. Lynch

**Affiliations:** 1 Museum of Natural Science, Louisiana State University, Baton Rouge, LA, United States of America; 2 Department of Ecology and Evolutionary Biology, University of California, Los Angeles, CA, United States of America; 3 Department of Anthropology, Tulane University, New Orleans, LA, United States of America; 4 Department of Anthropology & Archaeology and Department of Medical Genetics, University of Calgary, Calgary, AB, Canada; 5 Alberta Children’s Hospital Research Institute, University of Calgary, Calgary, AB, Canada; 6 Bilingual Biology Program, Glendon College, York University, Toronto, ON, Canada; 7 School of Biology, University of Costa Rica, San José, Costa Rica; 8 Laboratory of Conservation Biogeography and Macroecology, Federal University of Pará, Belém, PA, Brazil; 9 Institute for Society and Genetics, University of California, Los Angeles, CA, United States of America; 10 Department of Anthropology, University of California, Los Angeles, CA, United States of America; Institute for Biological Research, University of Belgrade, SERBIA

## Abstract

The major histocompatibility complex (MHC) is an important gene complex contributing to adaptive immunity. Studies of platyrrhine MHC have focused on identifying experimental models of immune system function in the equivalent Human Leukocyte Antigen (HLA). These genes have thus been explored primarily in captive platyrrhine individuals from research colonies. However, investigations of standing MHC variation and evolution in wild populations are essential to understanding its role in immunity, sociality and ecology. Capuchins are a promising model group exhibiting the greatest habitat diversity, widest diet breadth and arguably the most social complexity among platyrrhines, together likely resulting in varied immunological challenges. We use high-throughput sequencing to characterize polymorphism in four Class II DR and DQ exons for the first time in seven capuchin species. We find evidence for at least three copies for DQ genes and at least five for DRB, with possible additional unrecovered diversity. Our data also reveal common genotypes that are inherited across our most widely sampled population, *Cebus imitator* in Sector Santa Rosa, Costa Rica. Notably, phylogenetic analyses reveal that platyrrhine DQA sequences form a monophyletic group to the exclusion of all Catarrhini sequences examined. This result is inconsistent with the *trans*-species hypothesis for MHC evolution across infraorders in Primates and provides further evidence for the independent origin of current MHC genetic diversity in Platyrrhini. Identical allele sharing across cebid species, and more rarely genera, however, does underscore the complexity of MHC gene evolution and the need for more comprehensive assessments of allelic diversity and genome structure.

## Introduction

The genes of the major histocompatibility complex (MHC) code for antigen presenting proteins that function in the adaptive immune response of vertebrates. The MHC’s effectiveness stems from its polygenic and polymorphic nature; there are typically multiple gene copies, each with several alleles, thereby increasing the diversity of antigen-binding molecules available for recognizing potential threats to the organism. There are two principal classes of MHC genes: Class I (e.g., A, B, C) and Class II (e.g., -DP, -DQ, -DR). The proteins encoded by MHC Class II genes have an alpha and beta domain, each of which is coded by a separate gene (e.g., DQA and DQB, respectively) [1].

Much of our understanding of the MHC stems from the extensive immunological research on the equivalent Human Leukocyte Antigen (HLA), which has fueled explorations of MHC diversity and evolution in non-human primates, particularly in the more closely related catarrhines. Nonetheless, Platyrrhini, primarily from the families Callitrichidae and Aotidae, have contributed to models of human immune system function and to the understanding of MHC evolutionary history [2–5]. Such studies of MHC polymorphism have broader implications for our understanding of primate biology. For instance, evidence of non-neutral selection, including high d_N_/d_S_ ratios [6–8] and heterozygote advantage [9, 10], suggest that increased allelic diversity is beneficial and ultimately impacts individual fitness. Thus, studies of MHC in wild populations may also reveal functionally important ties to the evolutionary ecology of species [11] including signatures of selection related to parasitic infection dynamics [12, 13], mate choice [14–16] and kin recognition (see [17], for review).

Most platyrrhine studies are based on captive animals that experience limited mate choice and different immunological challenges compared to wild populations. The results of these studies vary, with extensive DRB sequence diversity found in *Saguinus oedipus* but generally low diversity for Class II MHC genes in callitrichid species [2, 18, 19]. MHC polymorphism data for other platyrrhine taxa are almost absent, typically limited to samples of one to three captive individuals to examine broader patterns of primate MHC evolution. While few studies have thoroughly examined wild populations of platyrrhines to shed light on MHC-related questions, the limited studies suggest a diverse and variable system. To date, studies of Class II MHC in wild populations of platyrrhines have revealed some diversity in the DP and DQ genes, and extensive variation in DR genes (Aotidae [5, 20–23]; *Alouatta pigra* [24]).

In this study, we expand our knowledge of platyrrhine MHC polymorphism by using primarily non-invasive sampling [25] and high-throughput genotyping of wild individuals [26, 27] from seven capuchin species (Platyrrhini: Cebidae: Cebinae). Among platyrrhines, this group arguably displays the greatest breadth of habitat types, diet diversity and social interactions and so presents a promising system for understanding adaptive MHC variation in the context of evolutionary ecology. Capuchins are omnivorous generalists that occupy and forage from forest floor to canopy and frequently use ground water sources [28]. Capuchins are also extremely social, characterized by large meta-populations with group sizes typically ranging from eight to 35 individuals, though *Sapajus flavius* groups can reach 90 individuals [29]. Capuchins also often form mixed species associations with other primates, especially in their Amazonian range [30]. Sociality can be hierarchical among males and females and additionally features parallel male dispersal across groups [31–34]. Such social behavior in animals can increase the occurrence of pathogen transmission [35, 36]. These primates also occupy a wide range of biomes extending from southern Central America through much of South America including the harsher dry savannah-like regions of the Llanos, Cerrado and Caatinga. Primate diversity in these latter regions is low relative to tropical forest regions likely due to the novel ecological challenges presented in these areas.

We comparatively evaluated MHC variation at multiple scales in capuchins by: (1) genotyping and comparing polymorphism in *Cebus imitator* individuals within and across Costa Rican populations; (2) comparing polymorphism across capuchin genera (*Cebus* and *Sapajus*) by providing data from limited sampling for six additional species–*C*. *olivaceus*, *C*. *albifrons*, *C*. *kaapori*, *Sapajus nigritus*, *S*. *xanthosternos* and *S*. *apella sensu lato* (recent molecular phylogenetic analyses support four monophyletic *Sapajus* species: *S*. *xanthosternos*, *S*. *robustus*, *S*. *nigritus*, and *S*. *apella* sensu lato [37] and accordingly we include *apella*, *cay*, *libidinosus* and *macrocephalus* morphospecies sampled here as part of *S*. *apella*); and (3) analyzing the broader evolution of these exons across Anthropoidea using a phylogenetic approach. We focus on Class II beta genes as these code for molecules that bind and present antigens of extracellular origin to their corresponding T cells [1] and beta-chain loci have been reported as more polymorphic than alpha-chain loci [38]. We expect that diversity in beta-chain genes would reflect responses to extrinsic immunological challenges presented by ecology and sociality in addition to providing information about the evolutionary history of primate MHC. Also, these genes have been examined across other primates providing data for comparison. Specifically, we chose the DR beta gene exon 2 (DRBe2) because (1) it is reported as the most variable exon among MHC genes, (2) it is the most widely studied exon across primates and (3) this exon contains the peptide binding region (PBR). The PBR (also known as the antigen-binding site, or ABS) allows antibodies to recognize and bind antigens of foreign organisms, a key process in the immune response [39]. Additionally, we chose exon 3 for DR beta (DRBe3), DQ alpha (DQA) and DQ beta (DQB) genes because exon 3 is not likely subject to convergent evolution, thus we expect it to reflect an accurate gene history [40, 41]. Furthermore, studies have shown that exon 3 may reflect more variability than exon 2 in these loci for platyrrhines [40, 42].

## Materials and methods

### Study sites and sampling

**Fig 1:**
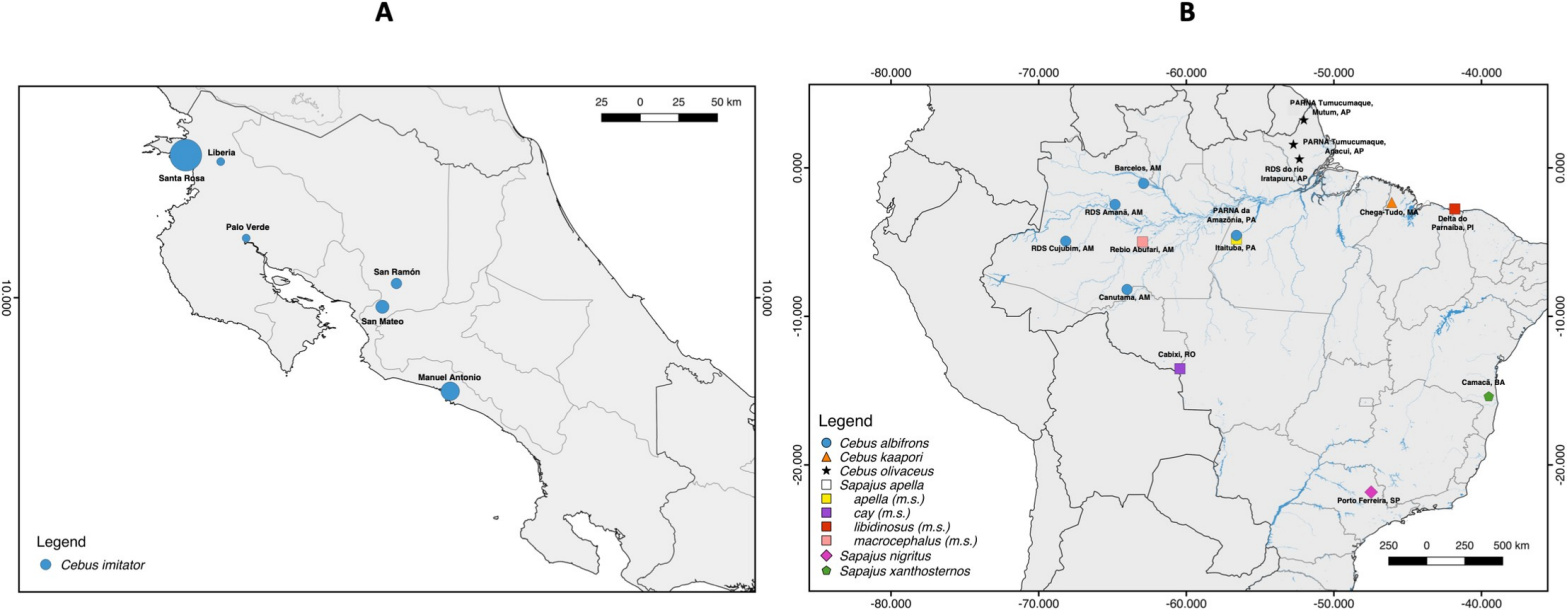
Sampling localities for capuchin species. (A) *Cebus imitator* sampling localities in Costa Rica. Circle size is proportionate to the number of individuals sampled at each location. (B) Sampling localities for the remaining cebine species evaluated in this study from South America. The legend shows the species demarcated by each symbol. “m.s” indicates morphospecies of *Sapajus apella sensu lato*.

Opportunistic non-invasive collections of fresh fecal samples from known *Cebus imitator* individuals residing in the Sector Santa Rosa (SSR) of the Área de Conservación Guanacaste, Costa Rica began in 2008 (Fig 1). The full SSR metapopulation is estimated at approximately 700 individuals in 48 groups, averaging 15 individuals per group [32]. This population has been the subject of long-term research since 1983 that includes pedigree data leveraged in this study. We successfully amplified and sequenced fecal DNA from 114 SSR individuals and tissue DNA from one individual that died of natural causes. This study was approved by Tulane University’s Animal Care and Use Committee (Protocol 0399R2). Samples were collected in accordance with the laws of Costa Rica, the United States, and Canada and complied with protocols approved by the Área de Conservación Guanacaste and by Tulane University’s Animal Care and Use Committee (Protocol 0399R2) and the Canada Research Council for Animal Care through the University of Calgary’s Life and Environmental Care Committee (ACC protocol AC15-0161). Samples were collected with permission from the Área de Conservación Guanacaste (ACG-PI-033-2016 and PI-063-2014) and CONAGEBIO (R-001-2015-OT-CONAGEBIO and R-025-2014-OT-CONAGEBIO). Samples were exported from Costa Rica under permits from the Área de Conservación Guanacaste (DGVS-030-2016-ACG-PI-002-2016; 012706) and imported to Canada with permission from the Canadian Food and Inspection Agency (A-2016-03992-4) and to the United States with permission from the Center for Disease Control (PHS Permit No. 2010-01-140).

Additionally, we extracted DNA from blood samples of 33 white-faced capuchins from five additional sites in protected and non-protected areas (San Ramón, Manuel Antonio, Liberia, San Mateo and Palo Verde) in Costa Rica (Fig 1A). These samples, maintained in a biological sample repository at the Universidad de Costa Rica, were collected for previous studies (see [43–45]) under the protocols established by the Animal Welfare Board (Comité Institucional para el Cuidado y Uso de los Animales) of the Universidad de Costa Rica and Dr. Gutierrez-Espeleta’s collections adhered to the legal requirements of Costa Rica (permit number 137-2010-SINAC). We also utilized blood and tissue samples from museum collections in Brazil for 24 other wild-caught individuals of known provenance representing six capuchin species (Fig 1B): *Cebus albifrons* (N = 12), *C*. *kaapori* (N = 1), *C*. *olivaceus* (N = 5), *Sapajus apella sensu lato* (N = 4), *S*. *nigritus* (N = 1), *S*. *xanthosternos* (N = 1). In total, we sampled 172 individual capuchins from seven species (note that there is taxonomic uncertainty in terms of species number in the genus *Sapajus*) [37]. Sample information is provided in S1 Table in S1 File. At UCLA, fecal samples were preserved at 4°C in RNAlater and blood and tissue samples were preserved at -80°C until extraction.

### DNA extraction and amplification

Genomic DNA was extracted from fecal, blood and tissue samples using the QIAmp DNA Stool Kit (Qiagen, Valencia, CA) and the Qiagen DNeasy Blood and Tissue Kit (Qiagen, Valencia, CA), respectively, following the manufacturer’s protocol with modifications from [46]. Resulting extracts were quantified using a Qubit dsDNA BR Assay (Life Technologies, Carlsbad, CA). For each sample, a 5-10uL aliquot of the extract was included in 25uL PCR reactions that included the following reagents: 12.5uL of PCR Master Mix (Promega, Madison, WI), 0.8uL of the forward primer (10uM), 0.8uL of the reverse primer (10uM), 0.8uL BSA and finally PCR-grade water to bring the final volume to 25uL. Primers were designed from alignments of published platyrrhine sequences, with preference for primers to match the *Cebus imitator* genome from GenBank (Table 1). PCR cycling conditions for all exons were: (1) an initial denaturing at 94°C for 2 minutes followed by (2) 35 cycles of: 94°C for 30 seconds, 58°C for 40 seconds and 72°C for 1 minute, then (3) a final extension at 72°C for 7 minutes. The PCR product was visualized on a 1.5% agarose gel to confirm the presence of fragments of the expected size.

**Table 1:** Exon sequence and primer information.

Exon	Exon Length	Recovered Length	Forward Primer Sequence	Reverse Primer Sequence
**DRBe2**	267	248	CGGATCGTTCGTGTYCCCACAG	CTCTCCGCTGCACTGTGAAGCT
**DRBe3**	282	214/217	TGACTGTGTATCCTGCMAAGACCCAG	ATTGCACTGTGRKAGGGCTCRTCA
**DQAe3**	279	229	CCTCACCRCAGAGGTTCCTGAGG	TCCAGGCCCCAGTGCTCC
**DQBe3**	279	254	GTCTTTCCCTGTCTGTTACTGCCCT	GTGATGGGGCTCTGGAGGCT

Exon length refers to the number of base pairs within the exon. Recovered length refers to the actual number of base pairs amplified from the primers used.

### Library preparation and sequencing

We purified the resulting amplicons to remove primer dimers and fragments less than 100bp by binding PCR products to 20uL of Serapure beads [47] and performing ethanol washes. DNA was subsequently eluted from the beads with 50uL of 10mM Tris pH 8.5. Products were again visualized on a gel to ensure removal of small fragments. Following PCR purification, we prepared libraries for sequencing by attaching unique dual index combinations to DNA from each individual using the Nextera XT indexing kit (Illumina, San Diego, CA), following the manufacturer’s protocol. We then purified the indexed amplicons using Serapure as before. Indexed amplicons were then quantified using a Qubit dsDNA BR Assay (Life Technologies, Carlsbad, CA), visualized using an Agilent Bioanalyzer (Agilent Technologies, Santa Clara, CA) and then pooled in equimolar ratios for 2x250 sequencing on an Illumina MiSeq machine (Illumina, San Diego, CA) at the Technology Center for Genomics and Bioinformatics (University of California, Los Angeles).

### Post-sequencing quality control

We began by separating the sequences of the four loci (DRB exon 2 and 3, DQA exon 3, DQB exon 3) based on their primer sequences. We then discarded any reads with primer sequences that differed from the original sequences by more than one base pair (bp). For the remaining reads, primer sequences were removed. We then merged the forward and reverse reads in Geneious Pro 9.0.5 [48] to get the full-length sequence. Only reads that were successfully paired with their partners were used in subsequent steps. Using Galaxy, we filtered out any reads shorter than 150bp or longer than 400bp and any reads where more than 5% of bp had a Phred score less than 20. The remaining reads were clustered into unique sequences using Geneious Pro 9.0.5 and singleton variants were discarded.

We concatenated all reads, labeled with the individual’s ID, into a single fasta file and aligned them using MAFFT 7.130b [49]. Subsequently, we separated reads by individual and corrected insertion/deletion homopolymer errors. We removed any variants that remained with incorrect reading frames; that is, any variants with lengths that differed from the expected exon length by any value that was not a multiple of 3.

As the number of MHC-DRB, DQB and DQA copies is unknown for capuchins, we assumed an average amplification efficiency of 0.7 and one gene copy (a maximum of 2 alleles) per individual while targeting at least three reads per allele. Therefore, individuals with less than 25 reads at the end of this process were discarded as having too few reads to determine a complete genotype at 99.9% confidence level [50]. After the initial genotyping (see below), most individuals had more than two alleles (averaging between 4–8 alleles per exon) so that the threshold for minimum number of reads to determine a complete genotype under these conditions increased to 120 [50].

### Genotyping

**Fig 2:**
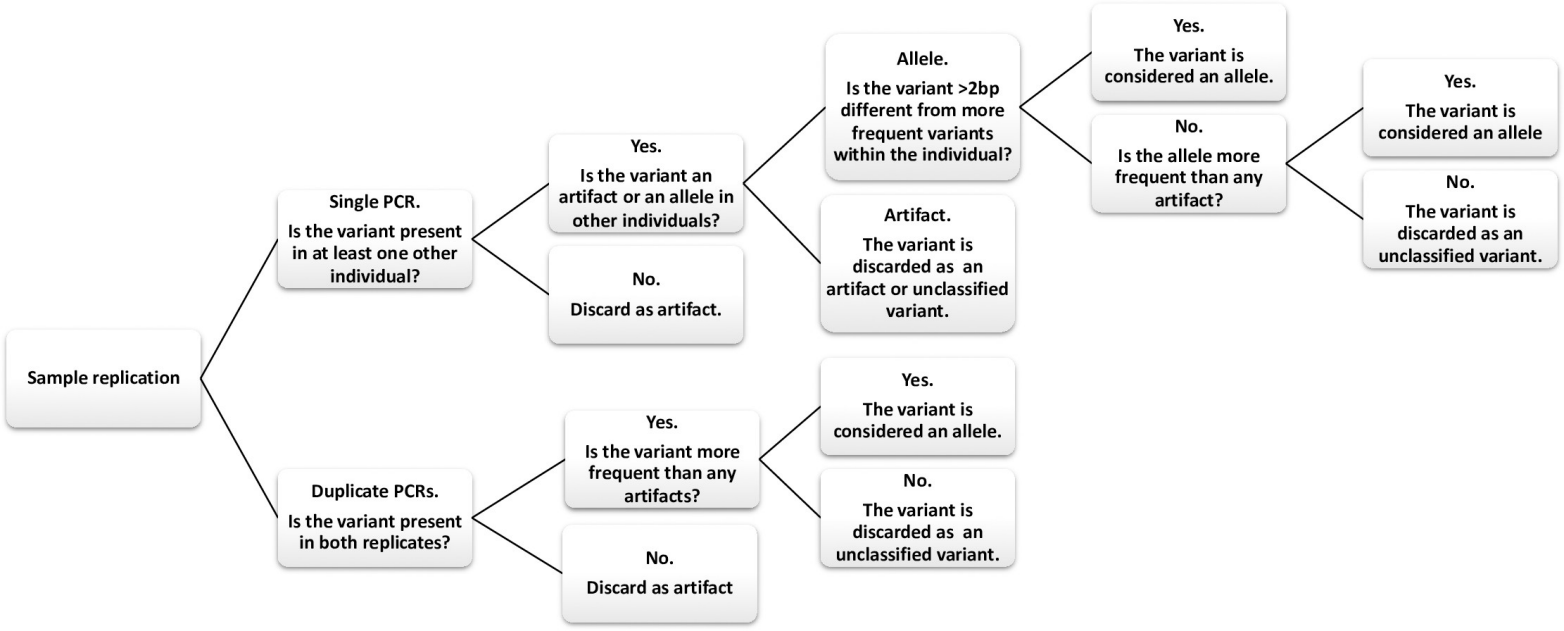
Genotyping decision tree. Sequence of steps used for distinguishing putative alleles from putative artifacts.

For *Cebus imitator*, we largely followed the genotyping steps for MHC loci outlined in [27]. For each individual, we sorted variants by their relative frequency. The most frequent variant was automatically considered a putative allele. Among the remaining variants, we discarded any that did not represent more than 1% of the total proportion of reads for the amplicon. We then verified that the remaining sequences were not chimeras using the *uchime2_denovo* command in UCHIME [51]. We discarded putative chimeras only if found to be a chimera in all individuals that the sequence occurred. The remaining variants were then sorted into the following two categories: (1) those with 1-2bp differences from any more frequent variant; (2) those with more than 2bp differences from any more frequent variant. We then distinguished between putative alleles and artifacts based on the decision tree illustrated in Fig 2.

Further quality control was adapted from [52] to graphically identify thresholds (based on the initial genotyping of *Cebus imitator* samples) to eliminate most of the artifactual alleles from PCR or sequencing errors from all samples (S1 Fig in S1 File). We assumed a minimum of two gene copies for all loci and our criteria for including alleles in the final analyses were that they (1) must have a mean relative species frequency of at least 0.07 across all individuals with the allele; (2) must have a relative frequency of at least 0.04 within the individual of interest. For DRBe3, we used a relative frequency threshold of 0.025 within the individual because the high copy number in this exon led to lower frequencies per allele, especially in heterozygotes with up to 10 alleles. If the allele was not one of the four most frequent alleles for the individual, it must also have a DIS score greater than one from any of the four most frequent alleles (DIS is smallest number of nucleotide differences from the four most common alleles of an individual).

Due to the large sample size available for *Cebus imitator*, we further required at least two individuals meet all the above criteria for an allele to be kept as a true allele. At the end of genotyping, there was a single allele which occurred in several *imitator* individuals that differed by one base pair from the most frequent allele but was inconsistent in being within or outside the four most frequent alleles. As a result, this sequence was inconsistent with several parental-offspring triads and we decided to take a conservative approach and remove it from the dataset. For other capuchin species, if a sequence was the most frequent variant in an individual it was labeled an allele. For these less well sampled species, after following the quality control measures modified from [52], we kept alleles as ‘true’ even if they were only found in one individual. The final set of recovered alleles for all exons is available on GenBank (accession MW677630-MW677937).

As an additional quality control measure for our MHC dataset for the SSR population, we incorporated analyses of known parent-offspring triads. These triads were based on long-term life history data collection in conjunction with microsatellite genotyping and paternity analyses previously performed for this population [53]. Using the triad data, we could use expected inheritance patterns for the alleles in each of the four exons to biologically validate the allele sets recovered from our QC measures, with the expectation that each allele found in an offspring’s genotype would also be found in at least one of its parents. This type of segregation analysis for MHC Class II haplotypes has been successfully performed in parent-offspring triads for several studies on pedigreed captive macaques [54–56].

### Gene trees

We reconstructed phylogenetic trees to examine the evolutionary history of the DR and DQ exons in the context of Platyrrhini specifically and anthropoid primates in general. We aligned recovered alleles from all capuchin species in the study and from published DR or DQ sequences for other primates obtained from publications, Genbank and the Immuno Polymorphism Database for MHC in Non-human primates, including only single generic representatives for a given DRBe2 allele in Catarrhini and excluding some HLA-DRBe2 sequences that differed by only one base-pair, to reduce the size of the tree for computational tractability. For Platyrrhini, we included all unique sequences per species. When information was available to distinguish pseudogenes from published data they were excluded from the analyses. We performed analyses on the Cipres Science Gateway [57] in MrBayes 3.2.6 [58, 59] under two runs with four chains each (3 heated and 1 cold) for 50 million generations to reconstruct evolutionary relationships among sequences for DQA, DQB and DRBe3. For DRBe2, we ran the analysis for 150 million generations. For all exons, the data were divided by codon position into three partitions with gamma distributed rates and all possible nucleotide substitution models were explored for fit to the data (nst = mixed).

## Results and discussion

### Sequencing and genotyping

In this study, we present extensive new data for polymorphic MHC Class II DR and DQ exons for seven capuchin species. We amplified DNA extracted from fecal, blood and tissue samples and generated 19,640,172 raw reads on an Illumina MiSeq. Of these, 12,067,708 reads were exact matches to regions targeted by our primers. After quality control filtering, 6,290,266 reads (52%) were used to genotype individuals. All sequences retained after filtering were of the expected length (Table 1). Sequencing depth was sufficient to genotype all exons, but amplification and sequencing success varied across samples (S2 Table in S1 File). We found no significant relationship between the number of reads per individual and the number of alleles recovered per exon (S2 Fig in S1 File). The only exception was a weak correlation for DQA exon 3, but this appears to be driven by a small number of outliers with exceptionally large numbers of reads. Even these outliers fell within the range of 3–6 alleles per individual, as did most of the sampling regardless of the number of reads.

In total, we genotyped all four exons in 123 *Cebus imitator* individuals (at least one exon for 147 *C*. *imitator* individuals) and for all 24 individuals representing the remaining six capuchin species (Table 2). We produced duplicate PCRs of all exons for five *C*. *imitator* individuals as a quality control measure to assess prevalence of sequence artifacts. The number of alleles recovered in these five replicated individuals was consistent with SSR *C*. *imitator* ranges and averages for all exons following quality control measures that do not rely on producing duplicate PCRs (Table 3).

**Table 2:** Number of unique alleles (and amino acid) sequences recovered.

All *Cebus* (N = 4 species)	DQA	DQB	DRBe2	DRBe3
*Cebus imitator* (N = 147*)	10 (9)	13 (12)	14 (14)	24 (21)
Santa Rosa (N = 110*)	6	6	10	16
Manuel Antonio (N = 19)	7	7	7	11
San Ramon (N = 3)	7	9	4	11
San Mateo (N = 7)	7	9	6	14
Liberia (N = 2)	4	4	7	12
Palo Verde (N = 2)	6	7	4	10
PSM	6.2	7	6.3	12.3
MAA/locality	0.8	1.2	1.6	2.4
***Cebus albifrons* (N = 12)**	17 (11)	23 (23)	32 (30)	26 (18)
Canutama (N = 1)	5	5	3	7
Barcelos (N = 3)	9	11	11	7
Maraa (N = 3)	7	9	12	8
Itaituba (N = 1)	6	4	2	6
Jutai (N = 4)	9	10	11	14
PSM	7.2	7.8	7.8	8.4
MAA/locality	2	4	6	4.5
***Cebus kaapori* (N = 1)**	5 (5)	4 (4)	2 (2)	5 (5)
***Cebus olivaceus* (N = 5)**	10 (8)	12 (10)	8 (8)	15 (12)
Iratapuru (N = 2)	6	6	3	9
Mutum (N = 1)	4	3	4	9
Anacuí (N = 2)	9	7	5	8
PSM	6.3	5.3	4	8.7
MAA/locality	2	4	2	2
**All *Sapajus* (N = 3 species)**				
***Sapajus apella sensu lato* (N = 4)**	6 (5)	5 (5)	6 (6)	10 (8)
morphospecies *S*. *apella*	3	3	4	4
morphospecies *S*. *cay*	3	3	3	4
morphospecies *S*. *libidinosus*	3	3	2	4
morphospecies *S*. *macrocephalus*	4	3	1	3
***Sapajus nigritus* (N = 1)**	5 (5)	5 (5)	3 (3)	4 (4)
***Sapajus xanthosternos* (N = 1)**	6 (6)	5 (5)	3 (3)	5 (5)

Total number of unique alleles (and unique amino acid sequences) recovered for each capuchin species, and unique alleles per locality. For species with multiple sites sampled, per site means (PSM) and mean additional unique alleles added (MAA) per locality are also reported. These values likely represent the minimum diversity for each of these species and localities. Some individuals did not amplify for all markers, and thus were excluded from those markers.

**Table 3:** Mean and total numbers of MHC alleles per individual with duplicate PCRs.

	DQA	DQB	DRBe2	DRBe3
***Cebus imitator***	3.6	3.6	4	8.2
Individual 1	4	4	5	10
Individual 2	4	4	5	10
Individual 3	3	3	2	6
Individual 4	3	3	3	5
Individual 5	4	4	5	10

### Capuchin MHC class II polymorphism supports minimally three DQA, DQB and DRB gene copies

**Fig 3:**
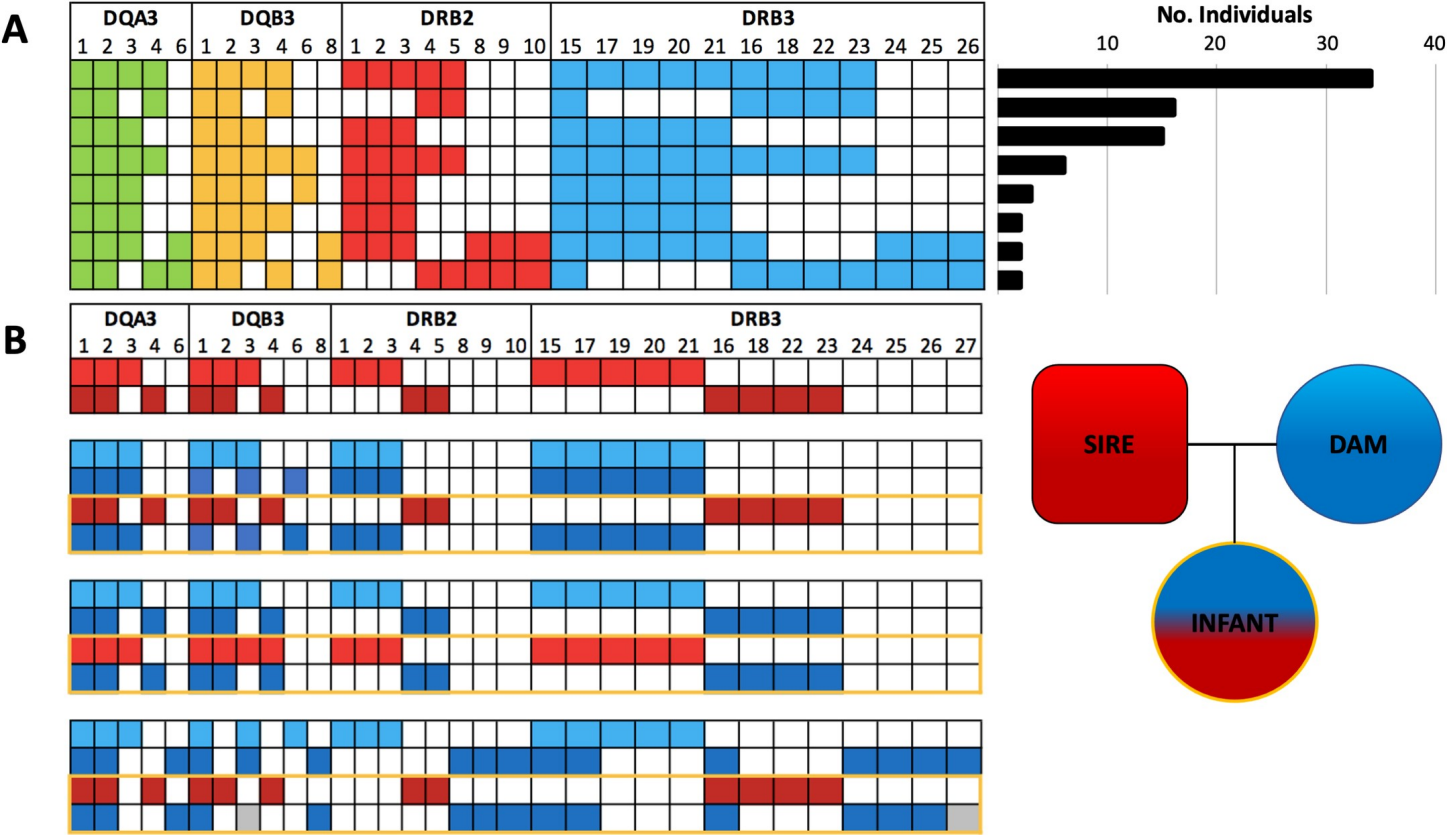
Genotype variation in the SSR population of *Cebus imitator*. (A) Diploid genotypes shared by two or more individuals. Only individuals successfully genotyped for all four exons are included (N = 97). (B) Illustration of genotype inheritance in the SSR *C*. *imitator* population based on our MHC genotypes as compared to known pedigrees in the population. Diploid genotypes are shown for three triads: A single male sire, three females with whom he sired offspring, and the respective resulting offspring. The two different shades of red (for the male) and blue (for the females) in the parental genotypes represent each individual’s two distinct haplotypes (which are identical if the individual is homozygous). Offspring genotypes (within yellow outline) are combinations of inherited paternal (red) and maternal (blue) haplotypes. Genotyping based on our quality control measures almost always shows expected patterns of inheritance that are consistent across all four exons (i.e. allele sets across all four exons are inherited together as a ‘block’). Gray boxes indicate potential allelic dropout due to failed amplification during PCR or erroneous removal during quality control.

We examined variation at multiple taxonomic scales which revealed patterns of allelic diversity and organization in platyrrhine MHC. Both population and species-level data suggest at least three copies of both DQA and DQB genes. We present evidence of this with pedigree data for the first time: inheritance patterns inferred from parental-offspring triads of the *Cebus imitator* Sector Santa Rosa population (SSR). Within SSR, we observed several genotypes that were found across multiple individuals (Fig 3A). Eighty individuals shared a four-exon genotype with others, with the most common genotype shared by 34 individuals (Fig 3A). Using the available pedigree data for SSR (see Methods) on parent-offspring triads, we inferred maternal and paternal MHC haplotype contribution to each offspring’s genotype across the four exons. This also allowed us to distinguish homozygous and heterozygous individuals for 35 known mother-father-offspring triads in the SSR data set (Fig 3B). Due to the presence of alleles that were fixed in the SSR population, and always present in both the parents and the offspring, we could determine that some gene copies had fixed alleles (always homozygous), while other gene copies for the same exon had more variation in alleles within the population. Among the 100 SSR individuals with four exons sequenced, we estimate that 33 were MHC homozygotes that inherited the same MHC haplotype across all four exons from both parents. Fifty individuals were heterozygous at all four exons, and fourteen additional individuals in SSR were homozygous for some exons but heterozygous for other exons. Overall, haplotype and genotype results were consistent with a minimum of three copies in the SSR population for all genes examined.

Evaluation of additional *Cebus imitator* populations increased the number of recovered alleles for all exons (Table 2). These *C*. *imitator* DQ and DR alleles consistently BLAST to multiple mRNA transcript sequences and to three sets of scaffold coordinates in the published *C*. *imitator* reference genome with 100% sequence identity (S3 Table in S1 File). Additionally, several capuchin species in our dataset average more than four DQ alleles per individual, again corroborating minimally three gene copies (Table 4). In previous studies, five DQA alleles and six DQB alleles were reported from just one *Saguinus oedipus* individual [40] and five DQB alleles in an *Aotus nancymaae* individual were confirmed [20]. Thus, the presence of a third set of DQ genes has now been demonstrated in three platyrrhine families despite the continued emphasis of two DQ copies that are orthologous with hominids [60, 61]. Understanding whether these additional DQ gene copies are functional or pseudogenes will require more genetics research.

**Table 4:** Mean number of MHC alleles per individual, by population, species and genus.

	DQA	DQB	DRBe2	DRBe3
**All *Cebus* (N = 4 species)**	4.5	3.9	3.4	7.5
*Cebus imitator* (N = 147*)	3.7 (2–6)	3.8 (3–6)	4 (2–6)	7.7 (3–10)
Santa Rosa (N = 110*)	3.6 (2–5, 4)	3.7 (3–5, 4)	4 (2–6, 5)	7.2 (3–10, 9)
Manuel Antonio (N = 19)	4.4 (3–5, 5)	4.3 (3–5)	4.4 (2–5, 5)	6.4 (3–9, 7)
San Ramon (N = 3)	4.0 (3–5)	5.0 (4–6)	2	7.0 (5–8, 8)
San Mateo (N = 7)	3.7 (3–6, 4)	4.3 (3–6, 4)	3.0 (2–4, 3)	7.3 (3–9, 8)
Liberia (N = 2)	3.5 (3–4)	3.5 (3–4)	5	9
Palo Verde (N = 2)	4.5 (4–5)	5	3.5 (3–4)	7.5 (7–8)
*Cebus albifrons*	4.8 (3–6, 5)	4.2 (3–6, 4)	4.3 (2–6, 4)	6.1 (5–8, 5)
Canutama (N = 1)	5	5	3	5
Barcelos (N = 3)	4.3 (3–5, 5)	4.6 (3–6)	5.3 (4–6, 6)	5.7 (5–7, 5)
Maraa (N = 3)	4.3 (3–5, 5)	4.3 (4–5, 4)	5.3 (4–6, 6)	6.0 (5–8, 5)
Itaituba (N = 1)	6	4	2	6
Jutai (N = 4)	5 (4–6, 5)	3.5 (3–4)	3.8 (3–4, 4)	6.25 (5–8, 6)
*Cebus kaapori* (N = 1)	5	4	2	5
*Cebus olivaceus* (N = 5)	4.4 (4–5, 4)	3.6 (3–5, 3)	3.2 (2–5, 2)	7 (6–9)
Iratapuru (N = 2)	4	4.0 (3–5)	2.5 (2–3)	6.5 (6–7)
Mutum (N = 1)	4	3	4	9
Anacuí (N = 2)	5	3.5 (3–4)	3.5 (2–5)	6.5 (6–7)
**All *Sapajus* (N = 3 species)**	4	3.7	2.7	4
*Sapajus apella sensu lato (N = 4*)	3.25 (3–4, 3)	3	2.5 (1–4)	3.75 (3–4, 4)
morphospecies *S*. *apella*	3	3	4	4
morphospecies *S*. *cay*	3	3	3	4
morphospecies *S*. *libidinosus*	3	3	2	4
morphospecies *S*. *macrocephalus*	4	3	1	3
*Sapajus nigritus* (N = 1)	5	5	3	4
*Sapajus xanthosternos* (N = 1)	6	5	3	5

Ranges and modes (if applicable) of numbers of alleles per individual shown in parentheses for populations and species with (N>1). Some individuals did not amplify for all markers, and thus were excluded from those markers.

Based on average per individual DRBe3 allele diversity, there appears to be at least five DR gene copies in capuchin individuals (Table 4). The diversity of DRBe3 alleles recovered generally exceeds that of DRBe2 both in the number of total alleles recovered per capuchin species, and the average number of alleles found per individual (Tables 2–4). This may be driven by multiple factors, but one possibility is that true pseudogenized exon 2 alleles were discarded during our QC while maintaining their corresponding exon 3 sequences that lacked indicative stop codons or frameshift indels. In the *Sapajus apella* genome (GCF_009761245), one sequence (XR_004266583.1) is a pseudogene with a 6 bp deletion and premature stop codon for DRBe2 but contains a DRBe3 identical to one of our *Sapajus* alleles. This parallels the pairing of a pseudogenized DRBe2 allele (XR_001823247.1) in the *Cebus imitator* genome (GCF_001604975.1) [62] with an apparently normal DRBe3 allele recovered in our study. Thus, based on our data and evidence from published genomes, it appears that while multiple and possibly variable numbers of copies of DR genes likely exist in capuchin species, not all of these are functional. Relatedly, it is possible that some of the gene copies of the capuchin MHC-DR locus are gene fragments that lack one or more exons, as seen in some MHC Class I and II gene copies [63–66].

Alternatively, PCR amplification of DRB exon 3 sequences could be more effective given our primer design and the nature of variation in exon 2 versus exon 3 sequences.

For instance, we used primers with degenerate bases to maximize the true alleles amplified in our PCRs. However, DRB exon 2 sequences appear to share less sequence identity than exon 3 sequences (Table 5). Therefore, it may be unlikely we amplified all existing diversity at exon 2 with a single pair of primers, even with degenerate bases. So, if DRB exon 3 sequences share more identity and our primers are more efficient in this case, it could explain differences in the recovered diversity for DRB exon 2 versus exon 3. Allelic dropout during PCR is also possible as we employed largely non-invasive sampling of feces for the majority of individuals sampled. Allelic dropout may have occurred for all four exons but may have been particularly problematic for DRB exon 2 in combination with reduced effectiveness of PCR amplification.

**Table 5:** Summary of MHC diversity by exon.

Metric	DRBe2	DRBe3	DQBe3	DQAe3
No. unique DNA alleles across capuchins	61	63	46	44
DNA alleles per individual across capuchins (mean and range)	3.9 (1–6)	5 (2–10)	3.9 (3–6)	3.8 (2–6)
No. unique a.a. alleles across capuchins	57	43	37	25
% polymorphic a.a. sites across capuchins	60%	38%	34%	21%
% polymorphic a.a. sites per capuchin species	18–48%	22–36%	13–28%	8–19%
mean pairwise identity between a.a. alleles within capuchin species	~80%	~90%	~92%	~95%
proportion of shared amino acid polymorphisms (*Cebus* and *Sapajus*)	0.69	0.58	0.39	0.36
transgenus DNA allele sharing across capuchins	0	2	2	2
transpecies DNA allele sharing across *Cebus*	2	9	9	8
transpecies DNA allele sharing across *Sapajus*	1	4	2	4
% DNA alleles unique to single *C*. *imitator* population	50%	28%	15%	10%

Yellow = less diverse, Red = more diverse.

*a.a. refers to amino acid

Finally, we made what we consider conservative decisions during quality control and genotyping in order to maximize elimination of artifacts. This may have led to the exclusion of real alleles that were rare in the population/species or represented by low read coverage, as well as pseudogenes of unexpected base pair length. Therefore, our study describes minimum levels of allele diversity across these exons in capuchin monkeys. The observed differences in our data between the average number of DRB exon 2 and exon 3 alleles may be biologically real, but there are several methodological factors that also could explain this pattern, especially considering that most studies report the greatest diversity from DRB exon 2 (though see [40, 42] that also describe relatively higher exon 3 diversity for platyrrhines).

### Patterns of MHC class II variation and evolution vary across taxonomic scales

We evaluated the extent of allele sharing at various taxonomic scales, starting with comparisons of six populations of *Cebus imitator*. Despite the SSR *Cebus imitator* population having the largest sample size, it had the second lowest allele diversity (total number of unique alleles) for DQ exons but the highest allele diversity for DR exons across the six *C*. *imitator* populations analyzed. Most alleles were shared across two or more *C*. *imitator* populations, with 90% shared for DQA and only 50% for DRBe2 (S4 Table in S1 File). At the species level, we find moderate trans-species allele sharing across at least two *Cebus* species for DQA (8 alleles, or 30% of all alleles shared), DQB (9 alleles, 24%), and DRBe3 (8 alleles, 15%) but very few shared across species for DRBe2 alleles (2 alleles, 4%; S5 Table in S1 File). Within *Sapajus*, one (9% of all alleles shared) and two (15%) alleles were shared for DRBe2 and DQB respectively, and four alleles for both DQA (31%) and DRBe3 (28%) (S5 Table in S1 File). While allele sharing across populations and species within cebine genera was relatively common, allele sharing is rarer at higher taxonomic ranks, with none occurring for DRBe2 alleles (S5 Table in S1 File). Across Platyrrhini, allele sharing of DRBe2 also occurs across species within the genus *Aotus*, and one allele is shared between callitrichid genera *Callithrix* (L12478.1) and *Saguinus* (M76488.1). One DRBe3 allele is shared between cebid genera *Cebus* (Ceal-DRB_38) and *Saimiri* (XM_01332220.1). However, trans-genus allele sharing of DQA and DQB is restricted to *Cebus* and *Sapajus*, and none of the alleles evaluated are shared between Platyrrhini and Catarrhini.

**Fig 4:**
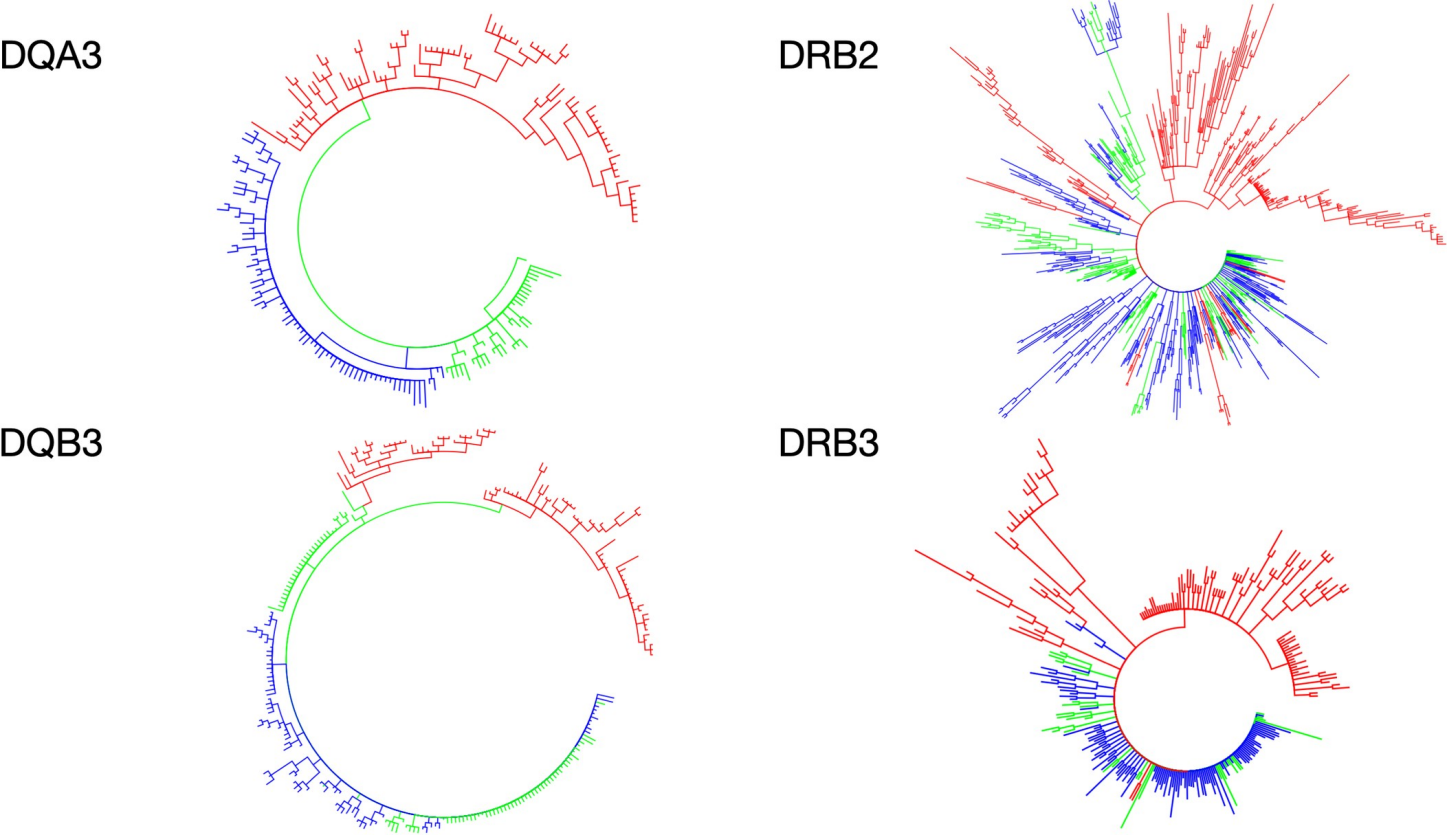
Polar gene trees. (A) DQA3, (B) DRB2, (C) DQB3 and (D) DRB3. Colors indicate major primate clades: Red–Platyrrhini; Blue–Cercopithecoidea; Green–Hominoidea.

When we consider our phylogenetic results (Fig 4, S3-S6 Figs in S1 File) trans-species polymorphism (TSP–identical, or nearly identical, sequences are maintained through speciation events, explaining allele sharing between variably related taxa [67] seems unlikely to explain patterns of MHC evolution across primates at the scale of infraorders. Most strikingly, Platyrrhini DQA alleles form a single well-supported clade (pp = 1) to the exclusion of Catarrhini (S5 Fig in S1 File). Similarly, early studies examining phylogenetic trees of primate MHC Class II gene sequences suggested that MHC variation largely originated after the divergence between Catarrhini and Platyrrhini lineages [68]. Monophyly of intron 2, exon 3 and exon 6 of platyrrhine DRB gene sequences suggested that the apparent shared ancestry among DRB exon 2 sequences between catarrhines and platyrrhines is a result of convergence on functionally important motifs in the PBR [41, 42, 69].

These interpretations are also consistent with phylogenetic analyses of exon 3 data from DQA, DQB, DPB and DRB from previous studies that overwhelmingly recover monophyly of platyrrhine sequences and thus argue for independent origins of functional MHC class II gene diversity in Platyrrhini [40]. So, together the data point to TSP being restricted to families or subfamilies within Platyrrhini. Examples of identical MHC allele sharing also occur in other sets of closely related primate taxa [5, 54, 70–73], mammals [74–76] and other vertebrates [77]. Thus, our study provides another dataset supporting the likely roles of both TSP and convergent evolution in the origin and maintenance of functionally beneficial motifs in MHC, perhaps acting at different taxonomic scales.

The action of complex genetic mechanisms on MHC loci, including the aforementioned, are such that reconstructing well-supported hypotheses of evolutionary history for these genes presents a persisting challenge [78]. While the few branches from the DRBe2 tree in which platyrrhines and catarrhines are found in clades together likely reflect convergence (see above), the short fragments from DRBe3 and DQB (214 and 254bp, respectively) provide little insight into the gene history. However, across the four exon trees, relative branch lengths reveal a pattern of limited differentiation across catarrhine alleles, and particularly limited differentiation in the hominoids, that contrasts with more extensive differentiation of alleles in Platyrrhini. This increased diversity may reflect a response to diverse selection pressures from new immunological challenges that early platyrrhines faced upon their arrival to and expansion across a new continent, South America.

Broadly, the fourteen alleles recovered from 140 *Cebus imitator* individuals for DRBe2, the most widely studied exon, seems to fall below the range of platyrrhine studies with similar sample sizes (Table 6). This is likely the result of sampling strategy; whereas other studies randomly sampled and targeted unrelated individuals, our study focused largely on a single population with multiple sets of related individuals. For the other capuchin species with smaller sample sizes and greater geographic coverage, the number of recovered DRBe2 alleles fall within the range of those from platyrrhine studies with similar sampling (Table 6). However, direct comparisons are difficult to make given the variability in the applied sampling and sequencing methodologies across studies. Standardization of methods and sample sizes across future studies will aid in comparative examinations of MHC variation.

**Table 6:** Reported DRB exon 2 variation in New World monkeys.

Species	No. Sequences Reported	No. Individuals Sampled	Reference
*Alouatta pigra*	21	44	[24]
*Aotus nancymaae*	67	71	[5]
	34	15	[21]
	16	25	[79]
*A*. *nigriceps*	30	15	[5]
*A*. *vociferans*	13	10	[5]
	18	23	[79]
*A*. *trivirgatus*	6	1	[80]
	5	1	[81]
*Callicebus moloch*	13	2	[80]
	5	1	[42]
*Ateles belzebuth*	3	1	[81]
*Pithecia pithecia*	3	1	[81]
*Saguinus oedipus*	5	1	[81]
	16	6	[80]
	6	2	[42]
	29	13	[19]
*S*. *labiatus*	6	8	[82]
*Callithrix jacchus*	6	2	[80]
	21	25	[2]
	2	1	[42]
	21	35+	[18]
	14	49	[83]
	22	15	[54]
*Cebuella pygmaea*	2	1	[81]
*Saimiri sciureus*	11	3	[80]
*Sapajus* spp.	5	2	[80]
	3	1	[42]
*S*. *apella* sensu lato	6	4	This Study
*S*. *nigritus*	3	1	This Study
*S*. *xanthosternos*	3	1	This Study
*Cebus imitator*	14	140	This Study
*C*. *albifrons*	32	12	This Study
*C*. *olivaceus*	8	5	This Study
*C*. *kaapori*	2	1	This Study

For comparisons within Cebinae, we consider the recovered allele diversity for each exon in the context of the number of localities sampled per capuchin species because of the differences in sample size (see Fig 1). For example, we found a total of fourteen DRBe2 alleles for *Cebus imitator* with an average of six total alleles recovered per locality. So, assuming we would recover about six unique alleles sampling just one locality and considering we recovered eight more unique alleles by sampling an additional five localities, we gained on average 1.6 additional unique alleles per added locality (see MMA/locality in Table 2). We see a comparable average of two additional unique alleles per added locality for *C*. *olivaceus*. However, in *C*. *albifrons*, we gain an average of six additional unique alleles per added locality. Coupled with the higher number of alleles recovered (32 total), DRBe2 seems to be more variable in *C*. *albifrons* relative to other *Cebus* species. This is also the only species in the dataset where overall DRBe2 variation exceeds DRBe3 variation.

While allele diversity for DQ genes remains comparatively low in *Cebus imitator*, increased levels of diversity are similar for *C*. *olivaceus* and *C*. *albifrons*. *C*. *kaapori* values fall within the range of recovered alleles when comparisons are restricted to localities with equivalent sample size, except for DRBe3 (Table 2). Within *Sapajus*, species have similar numbers of alleles reported for DQ exons, but for DRBe3 it is highest in *S*. *apella*. Again, if we compare the average allele diversity per locality, variation at all exons seems higher in *Cebus* versus *Sapajus* (Table 2). However, allele diversity in DRBe2 and DQB is similar across genera when comparing only localities with equivalent sample size. The total number of unique alleles is still generally lower for *Sapajus* for per individual, per locality, and per genus averages (Tables 2 and 4).

When we look at average pairwise differences in amino acid sequences for DRBe2, though, dissimilarity is greater in *Sapajus* (22.8% mean dissimilarity) than in *Cebus* (16% mean dissimilarity). *Sapajus apella*, the species sampled across the most diverse habitats, has the greatest mean within-species amino acid sequence dissimilarity (24%) compared to the other sampled capuchin species in this study. The increased amino acid divergence in *Sapajus* is notable given the smaller sample sizes and lower number of total alleles recovered relative to *Cebus*. Perhaps *Sapajus* has responded to environmental drivers by diversifying DRBe2 in a limited number of highly distinct sequences, rather than by increasing the total number of alleles per se. In contrast, *Cebus* appears to have maintained an increased number of more similar alleles. Alternatively, neutral processes (e.g., drift) may explain these differences between independent lineages that might reflect divergent demographic histories.

**Fig 5:**
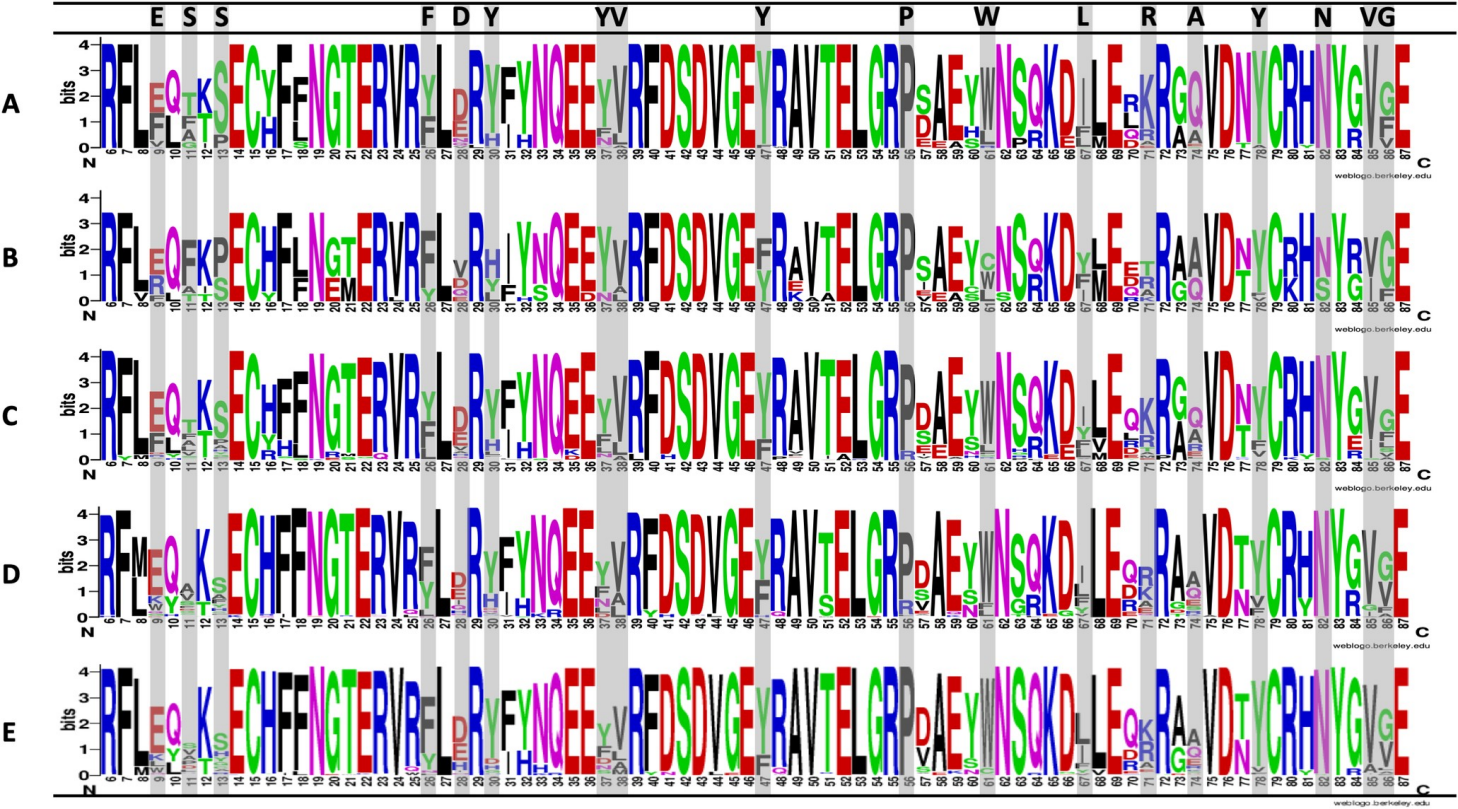
Graphical representation of the sequence conservation of amino acids (sequence logo) for DRB exon 2. Compares site variability between *Cebus* (A), *Sapajus* (B), Platyrrhini (C), Cercopithecoidea (D) and Hominoidea (E). Single letter codes are shown for amino acids and colors indicate their major biochemical properties: Red–acidic; Blue–basic; Green–polar; Black–hydrophobic. Generated using WebLogo [84]. Gray shading indicates predicted PBR (peptide binding region) sites based on human PBR in [39]. Letters in the top row indicate the most common amino acid at that site in human HLA alleles [39].

Within capuchins, there are also amino acid polymorphisms unique to only *Cebus* or *Sapajus* at twelve sites for DRBe2 (Fig 5). DRBe2 amino acid sequences had up to four variants at many sites within several capuchin species, and at some sites the most common amino acid varied across the species examined (see sites 4–8, 65, 66, 68, 69). Finding this signal in DRBe2 is not surprising considering DR genes are often the most variable MHC genes and exon 2, specifically, codes for the PBR, which has obvious functional importance in antigen recognition. Thus, DRBe2 allele variation in capuchins and other platyrrhines may be related to local adaptation to pathogens; in other words, there is stronger diversifying selection on DRBe2 than the other exons analyzed in this study (Table 5). Nearly all DRBe2 sequences feature substitutions that are non-synonymous while there is more redundancy in other exons (Table 2). Additionally, DRBe2 alleles were shared the least at all taxonomic scales (S4 and S5 Tables in S1 File). We also found that for DRBe2, there were three polymorphic sites in platyrrhines that were fixed across catarrhines (Fig 5). Platyrrhines had a different most common amino acid variant at five additional sites, as well as 26 sites with unique variants in platyrrhines not found in catarrhines. The DQ amino acid sequences were less variable across anthropoid primates than the DR sequences (see Fig 5, S7-S9 Figs in S1 File). So, while within population DNA variation was low relative to other exons, at higher taxonomic scales there is clearly more DRBe2 protein sequence diversity.

Our preliminary results demonstrate the promise of future studies leveraging analyses that directly evaluate potential relationships between selection and ecological characteristics. When we consider the diversity of exons at multiple levels of analysis, we see distinctly different patterns of sequence evolution. Sequence diversity and divergence increase with taxonomic rank, with the least diversity shown among related individuals of a single population. However, population data were vital to understanding MHC variation, as demonstrated by the confirmation of a third gene copy for both DQA and DQB using kinship information. Well-supported relationships in gene trees corroborate independent origins for platyrrhine and catarrhine MHC variation. Our study of multiple exons at varying phylogenetic scales revealed promising patterns that might reflect ecological signatures in an adaptive genetic locus. However, studies employing NGS methods should have at least two replicate sequences for all individuals when possible to avoid inflated or deflated allelic diversity. Although we mitigate this problem using alternative quality control methods that do not require duplicates, it is a limitation of our study that we could only produce replicates in five individuals. Additionally, to enable more quantitative analyses of selection and other potential processes in MHC across platyrrhine species, analyses of complete sequences from orthologous gene copies are needed.

## Supporting information

S1 File This file contains S1-S9 Figs and S1-S5 Tables. (PDF)
