# Supplementary material for: Major histocompatibility complex class II DR and DQ evolution and variation in wild capuchin monkey species (Cebinae)
Source: PLoS One. 2021 Aug 12;16(8):e0254604. doi: 10.1371/journal.pone.0254604 (PMC8360539; doi:10.1371/journal.pone.0254604)
Supplement: S1 File — (PDF) [file pone.0254604.s001.pdf]

S1 File

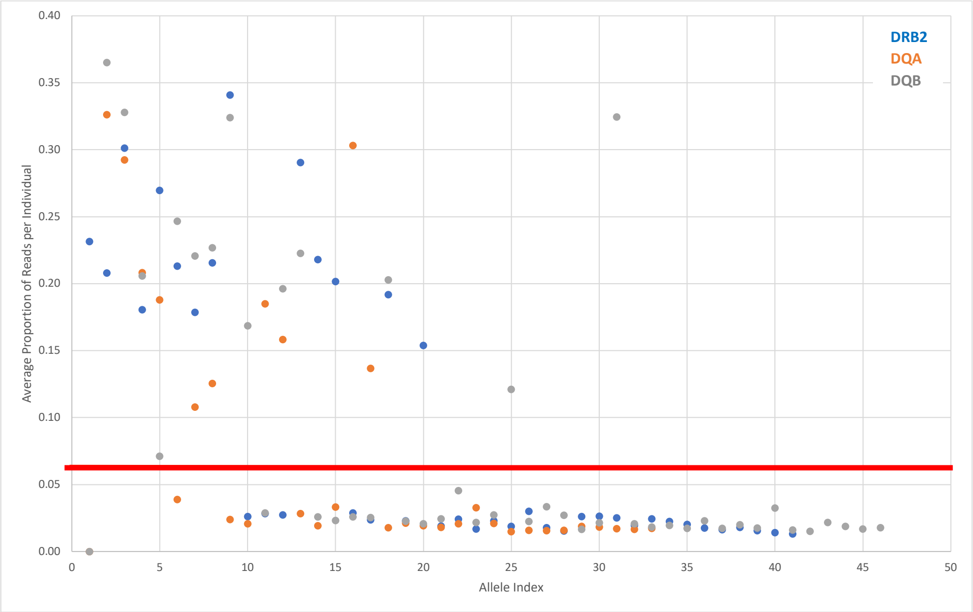

A

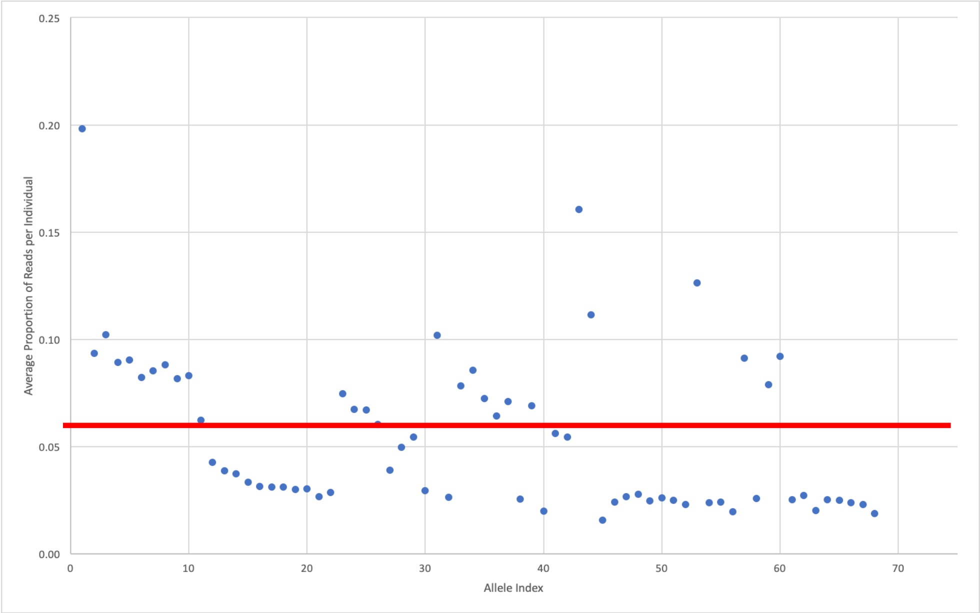

B

Supplemental Figure 1. Per-individual mean allele frequency (only includes *C. imitator* individuals for which the allele was present) plotted against allele rank (overall relative frequency in the data set) for all DRB exon two, DQA and DQB (A) and DRB exon three (B) alleles passing previous quality control measures. The threshold indicating true alleles (≥7%) versus artifacts (<7%) is illustrated by a red line.

Supplemental Information

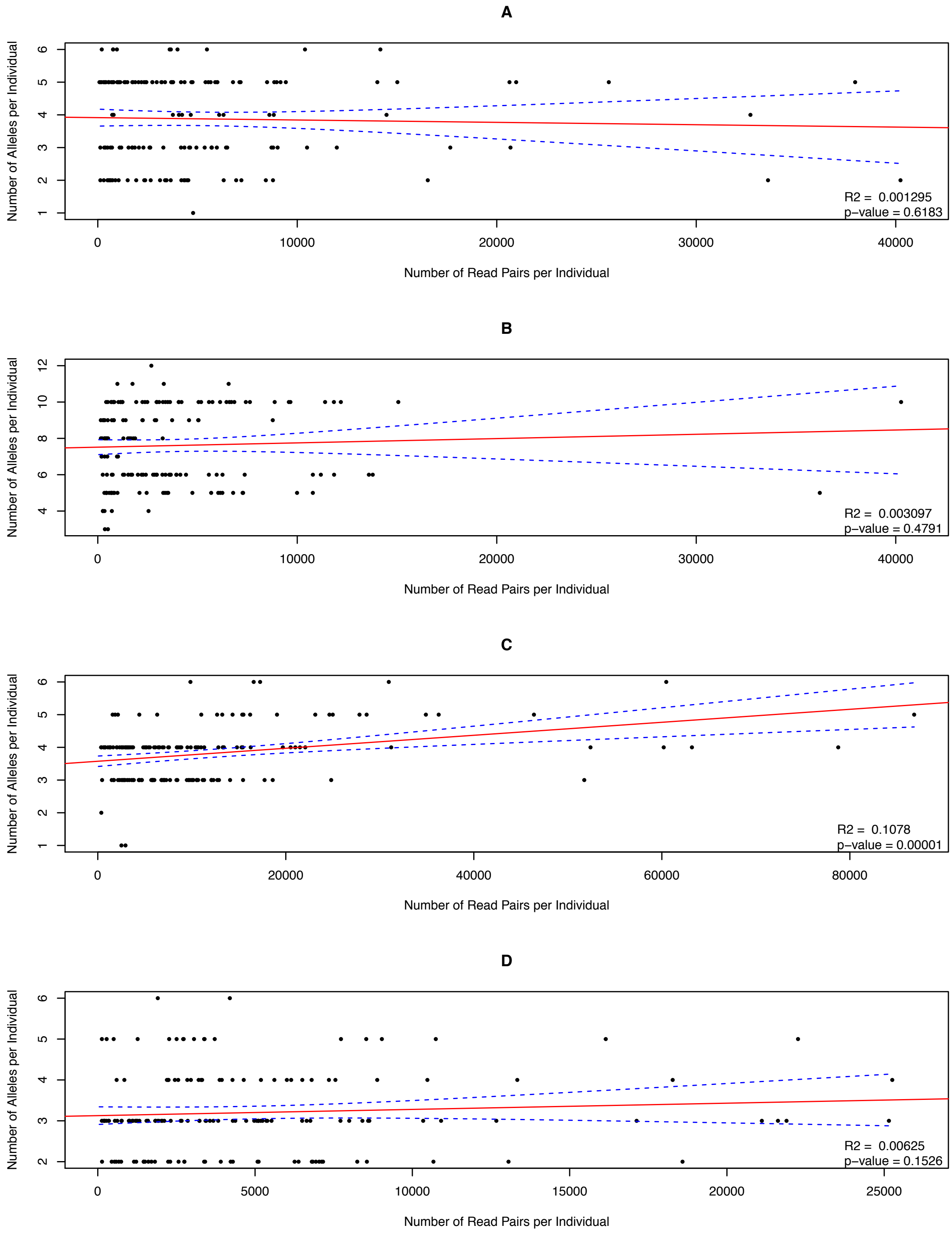

Supplemental Figure 2. Regression analyses of the number of read pairs per individual versus the number of alleles identified per individual. (A) DRBe2 (B) DRBe3 (C) DQAe3 (D) DQBe3. Dotted blue lines illustrate 95% confidence intervals.

Supplemental Information

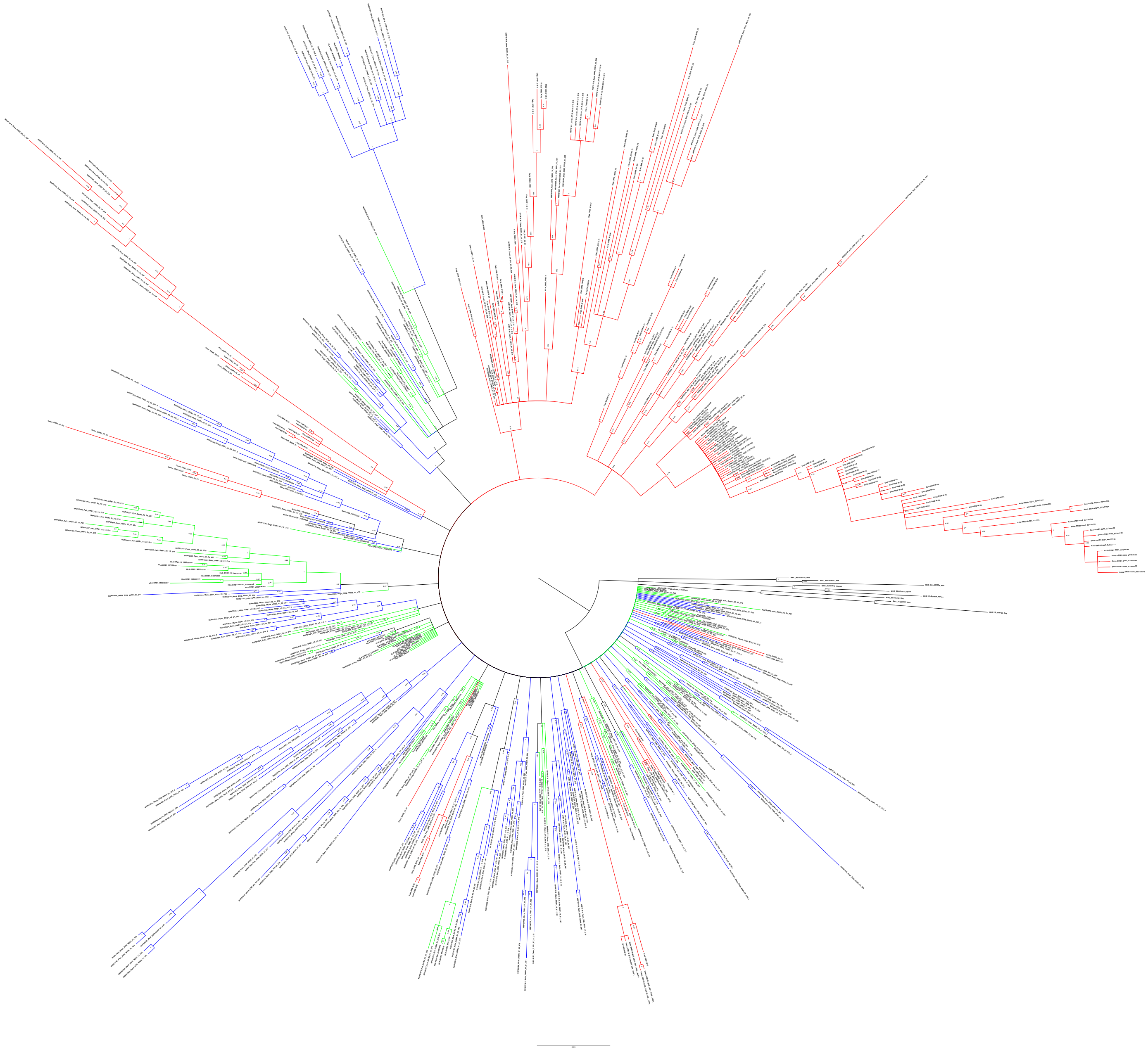

Supplemental Figure 3. Gene tree for DRB exon two sequences from across Primates and select mammalian outgroups (black clade). Colors indicate major primate clades: Red – Platyrrhini; Blue – Cercopithecoidea; Green – Hominoidea. Species are indicated by four letter abbreviations consisting of the first two letters of the genus and species names: Alpi = *Alouatta pigra*; Aoaz = *Aotus* azarae; Aona = *Aotus nancymaae*; Aoni = *Aotus nigriceps*; Aotr = *Aotus trivargatus*; Aovo = *Aotus vociferans*; Atbe = *Ateles belzebuth*; Caja = *Callithrix jacchus*; Camo = *Callicebus molloch*; *Capy = *Cebuella pygmaea*; Ceal = *Cebus albifrons*; *Ceap = *Sapajus apella*; Ceim = *Cebus imitator*; Ceka = *Cebus kaapori*; Ceol = *Cebus olivaceus*; Cepy = *Cebuella pygmaea*; Chae = *Chlorocebus aethiops*; Chsa = *Chlorocebus sabaeus*; *Coan* = *Colobus angolensis*; Gogo = *Gorilla gorilla*; HLA = *Homo sapiens*; Hymo = *Hylobates moloch*; Maar = *Macaca arctoides*; Mafa = *Macaca fascicularis*; Mafu = *Macaca fuscata*; Malo =

Supplemental Information

Macaca leonina; Mamu = *Macaca mulatta*; Mane = *Macaca nemestrina*; Masi = *Macaca silenus*; Masp = *Mandrillus sphinx*; Paan = *Papio anubis*; Papa = *Pan paniscus*; Patr = *Pan troglodytes*; Paur = *Papio ursinus*; Pipi = *Pithecia pithecia*; *Popy = Pongo pygmaeus*; Saap = *Sapajus apella*; Saca = *Sapajus cay*; Sali = *Sapajus libidinosus*; Sama = *Sapajus macrocephalus*; Sani = *Sapajus nigritus*; Saoe = *Saguinus oedipus*; Sasc = *Saimiri sciureus;* Saxa = *Sapajus xanthosternos*.
*Four letter codes for these species were assigned before recent taxonomic updates that lead to name changes.

Supplemental Information

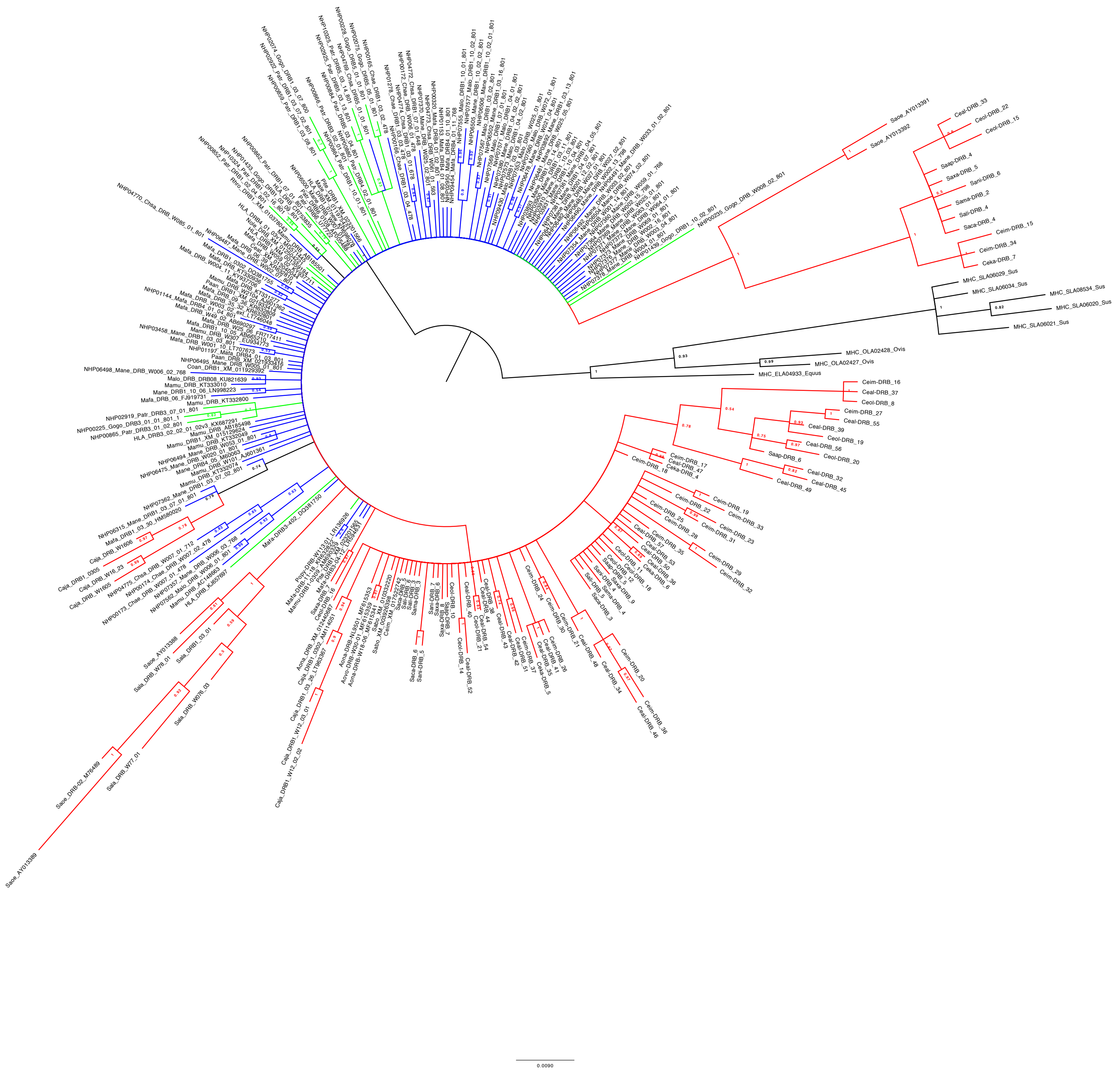

Supplemental Figure 4. Gene tree for DRB exon three sequences from across Primates and select mammalian outgroups (black clade). Colors indicate major primate clades: Red – Platyrrhini; Blue – Cercopithecoidea; Green – Hominoidea. Species are indicated by four letter abbreviations consisting of the first two letters of the genus and species names: Aona = *Aotus nancymaae*; Aovo = *Aotus vociferans*; Caja = *Callithrix jacchus*; Ceal = *Cebus albifrons*; Ceat = *Cercocebus atys;* Ceim = *Cebus imitator*; Ceka = *Cebus kaapori*; Ceol = *Cebus olivaceus*; Chae = *Chlorocebus aethiops*; Chsa = *Chlorocebus sabaeus*; Coan = *Colobus angolensis*; Gogo = *Gorilla gorilla*; HLA = *Homo sapiens*; Hymo = *Hylobates moloch*; Mafa = *Macaca fascicularis*; Mafu = *Macaca fascicularis*; Male = *Macaca leucophaeus*; Malo = *Macaca leonina*; Mamu = *Macaca mulatta*; Mane = *Macaca nemestrina*; Nole = *Nomascus leucogenys*; Paan = *Papio anubis*; Patr = *Pan troglodytes*; Paur = *Papio ursinus*; Pite = *Piliocolobus tephrosceles*; Popy = *Pongo pygmaeus*; Rhro = *Rhinopithecus roxellana*; Saap = *Sapajus apella*; Sabo = *Saimiri boliviensis*; Saca = *Sapajus cay*; Sali = *Sapajus libidinosus*; Sama = *Sapajus macrocephalus*; Sani = *Sapajus nigritus*; Saoe = *Saguinus oedipus;* Saxa = *Sapajus xanthosternos*.

Supplemental Information

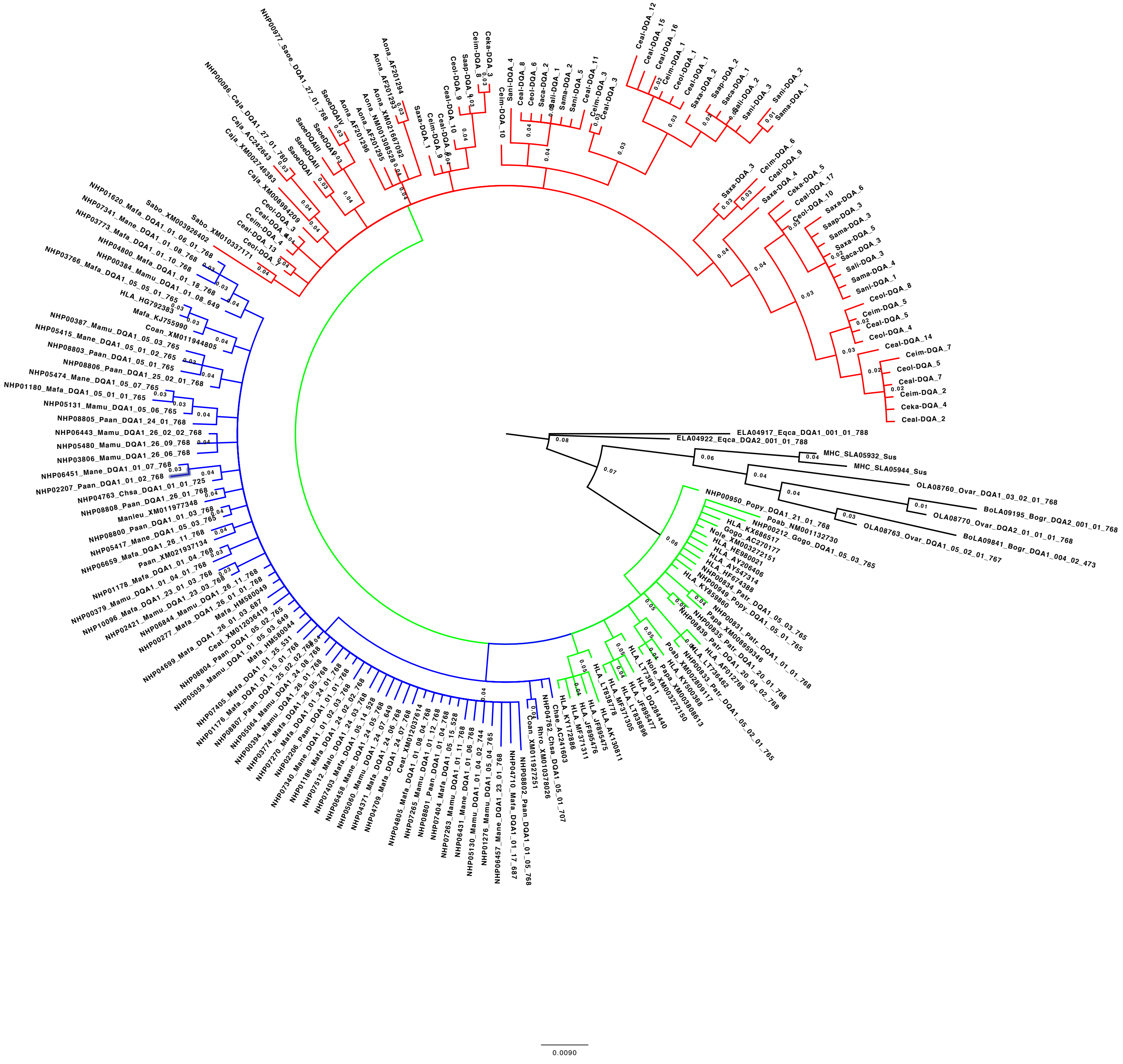

Supplemental Figure 5. Gene tree for DQA exon three sequences from across Primates and select mammalian outgroups (black clade). Colors indicate major primate clades: Red – Platyrrhini; Blue – Cercopithecoidea; Green – Hominoidea. Species are indicated by four letter abbreviations consisting of the first two letters of the genus and species names: Aona = *Aotus nancymaae*; Caja = *Callithrix jacchus*; Ceal = *Cebus albifrons*; Ceat = *Cercocebus atys*; Ceim = *Cebus imitator*; Ceka = *Cebus kaapori*; Ceol = *Cebus olivaceus*; Chae = *Chlorocebus aethiops*; Chsa = *Chlorocebus sabaeus*; Coan = *Colobus angolensis*; Gogo = *Gorilla gorilla*; HLA = *Homo sapiens*; Hymo = *Hylobates moloch*; Mafa = *Macaca fascicularis*; Malo = *Macaca leonina*; Mamu = *Macaca mulatta*; Mane = *Macaca nemestrina*; Nole = *Nomascus leucogenys*; Paan = *Papio anubis*; Papa = *Pan paniscus*; Patr = *Pan troglodytes*; Poab = *Pongo abelii*; Popy = *Pongo pygmaeus*; Rhro = *Rhinopithecus roxellana*; Saap = *Sapajus apella*; Sabo = *Saimiri boliviensis*; Saca = *Sapajus cay*; Sali = *Sapajus libidinosus*; Sama = *Sapajus macrocephalus*; Sani = *Sapajus nigritus*; Saoe = *Saguinus oedipus*; Sasc = *Saimiri sciureus;* Saxa = *Sapajus xanthosternos*.

Supplemental Information

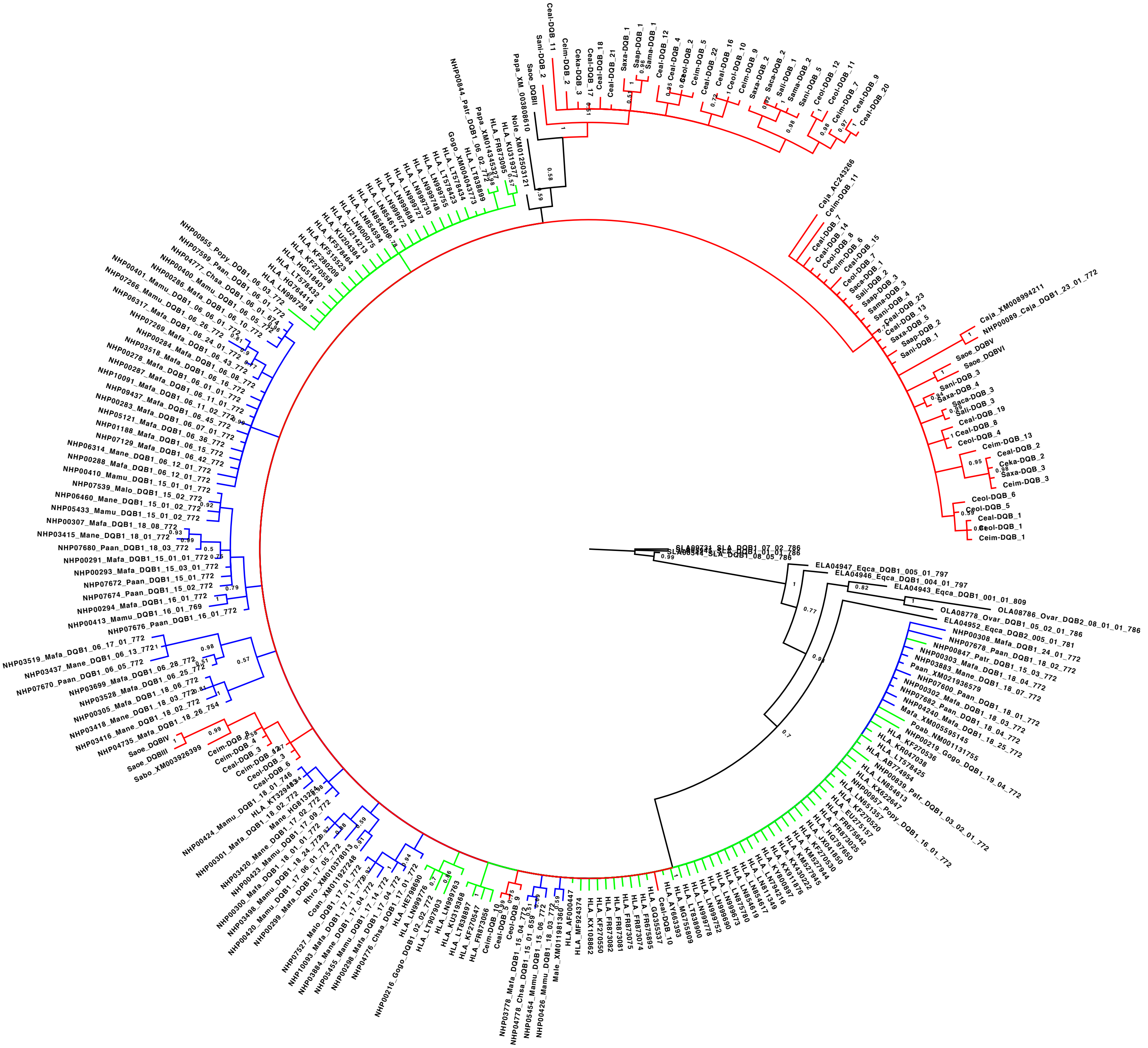

Supplemental Figure 6. Gene tree for DQB exon three sequences from across Primates and select mammalian outgroups (black clade). Colors indicate major primate clades: Red – Platyrrhini; Blue – Cercopithecoidea; Green – Hominoidea. Species are indicated by four letter abbreviations consisting of the first two letters of the genus and species names: Caja = *Callithrix jacchus*; Ceal = *Cebus albifrons*; Ceim = *Cebus imitator*; Ceka = *Cebus kaapori*; Ceol = *Cebus olivaceus*; Chsa = *Chlorocebus sabaeus*; Coan = *Colobus angolensis*; Gogo = *Gorilla gorilla*; HLA = *Homo sapiens*; Maar = *Macaca arctoides*; Mafa = *Macaca fascicularis*; Mafu = *Macaca fascicularis*; Male = *Macaca leucophaeus*; Malo = *Macaca leonina*; Mamu = *Macaca mulatta*; Mane = *Macaca nemestrina*; Nole = *Nomascus leucogenys*; Paan = *Papio anubis*; Papa = *Pan paniscus*; Patr = *Pan troglodytes*; Popy = *Pongo pygmaeus*; Rhro = *Rhinopithecus roxellana*; Saap = *Sapajus apella*; Sabo = *Saimiri boliviensis*; Saca = *Sapajus cay*; Sali = *Sapajus libidinosus*; Sama = *Sapajus macrocephalus*; Sani = *Sapajus nigritus*; Saoe = *Saguinus oedipus*; Sasc = *Saimiri sciureus;* Saxa = *Sapajus xanthosternos*.

Supplemental Information

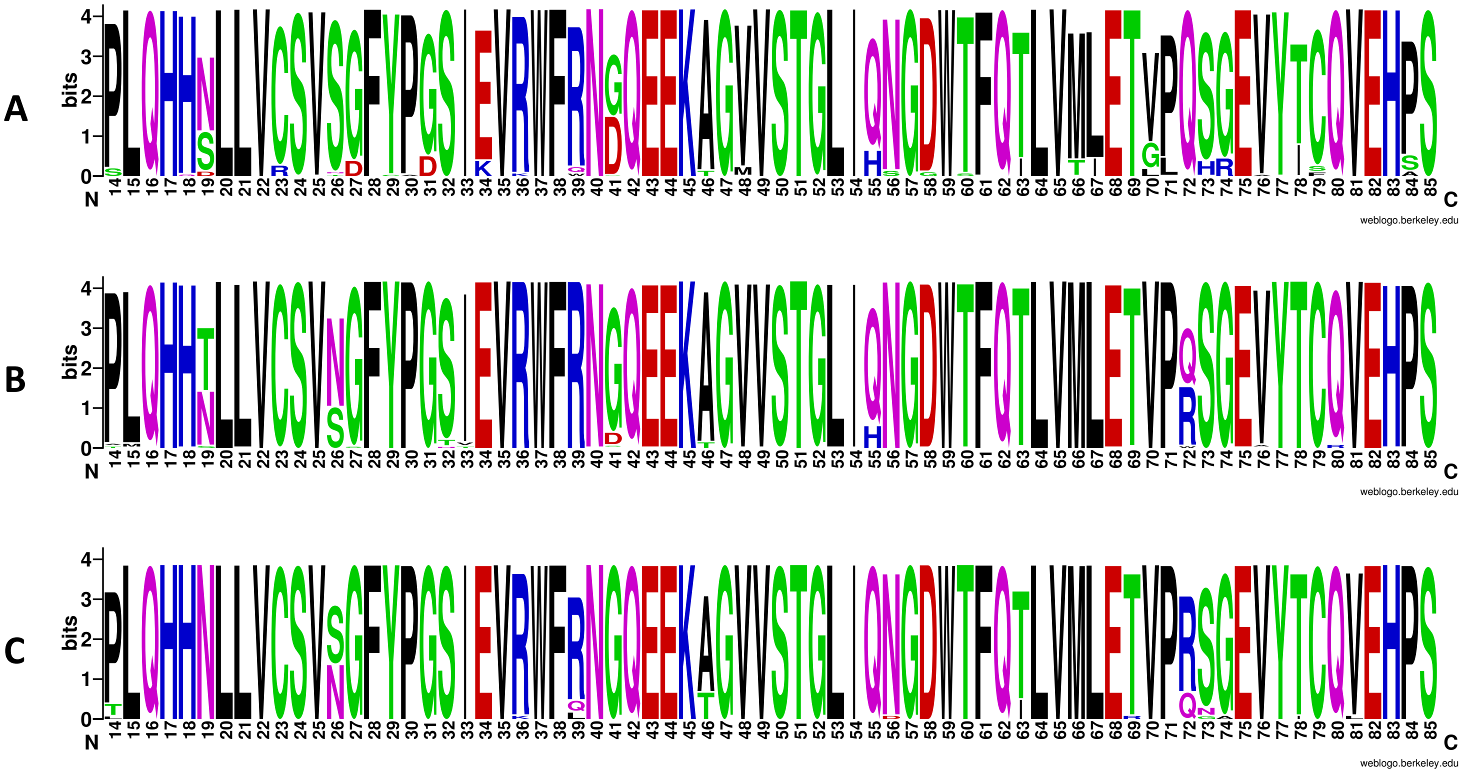

Supplemental Figure 7. Sequence logos generated using WebLogo (Crooks et. al., 2004) for DRB exon three comparing site variability between Platyrrhini (A), Cercopithecoidea (B), and Hominoidea (C). Single letter codes are shown for amino acids and colors indicate their major biochemical properties: Red – acidic; Blue - basic; Green – polar; Black – hydrophobic.

Supplemental Information

A

B

C

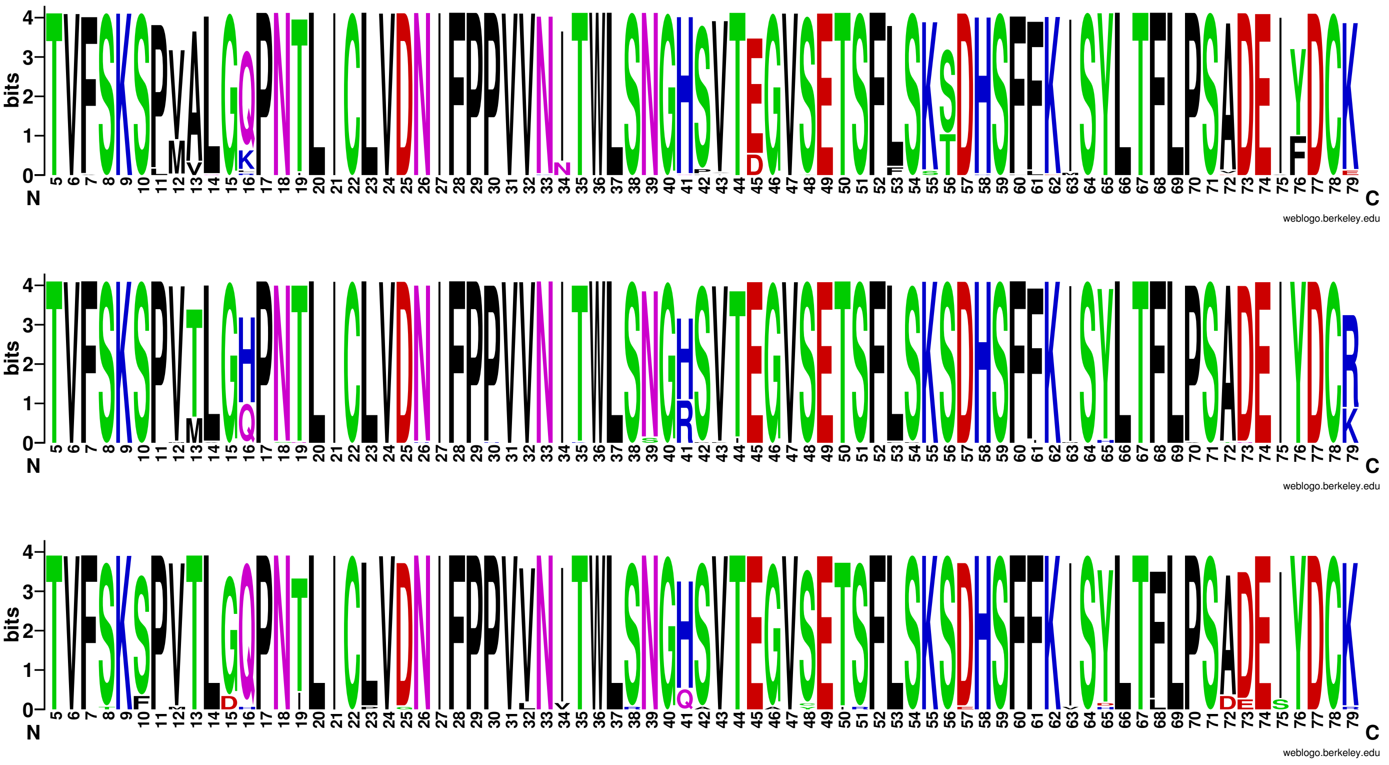

Supplemental Figure 8. Sequence logos generated using WebLogo (Crooks et. al., 2004) for DQA comparing site variability between Platyrrhini (A), Cercopithecoidea (B), and Hominoidea (C). Single letter codes are shown for amino acids and colors indicate their major biochemical properties: Red – acidic; Blue - basic; Green – polar; Black – hydrophobic.

Supplemental Information

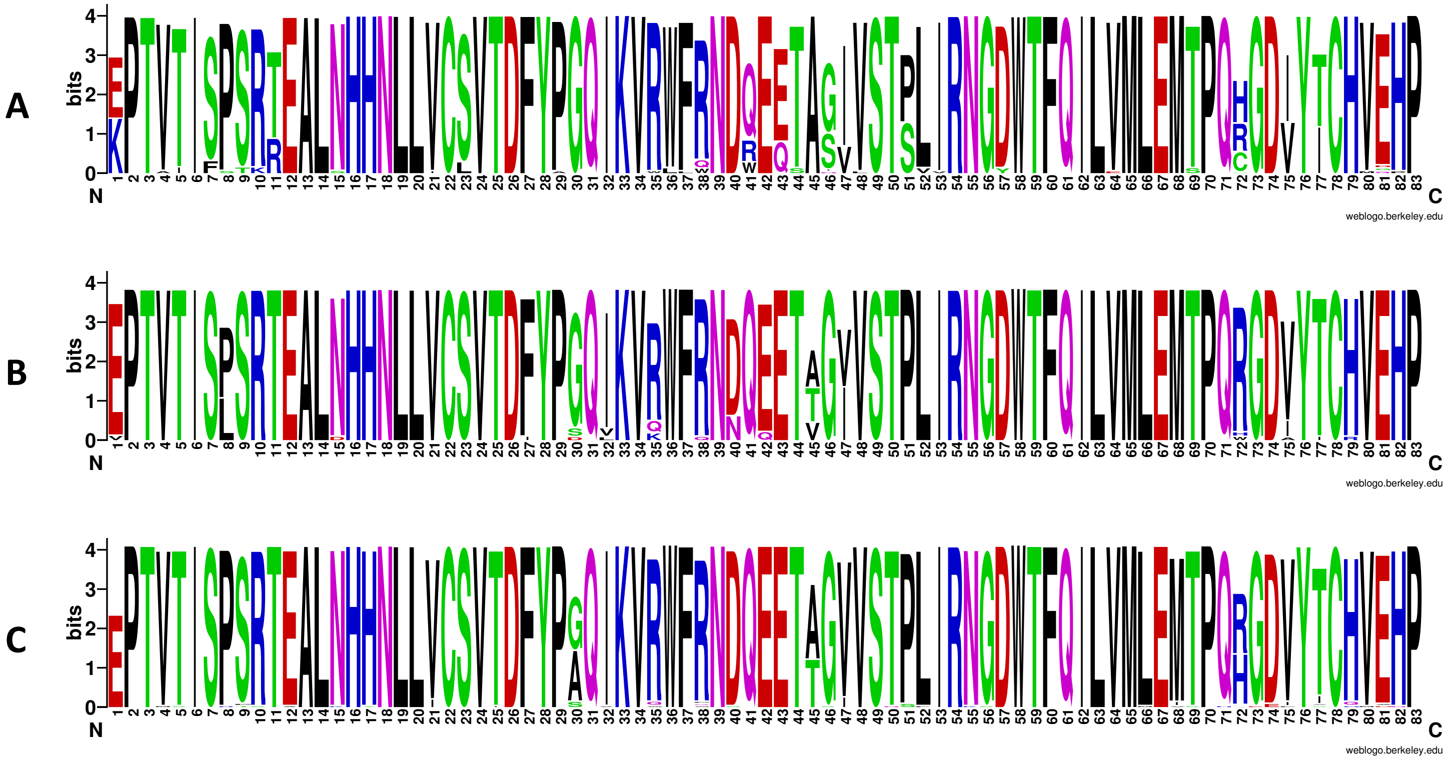

Supplemental Figure 9. Sequence logos generated using WebLogo (Crooks et. al., 2004) for DQB comparing site variability between Platyrrhini (A), Cercopithecoidea (B), and Hominoidea (C). Single letter codes are shown for amino acids and colors indicate their major biochemical properties: Red – acidic; Blue - basic; Green – polar; Black – hydrophobic.

Table S1. Sampling information for capuchin monkeys included in this study

**Individuals**	Species	Locality	Catalog No.	Material
Ceal_FR85	*C. albifrons*	Canutama, Amazonas	MPEG43805	Muscle
Ceal_IPB994	*C. albifrons*	Barcelos, Amazonas	MPEG43855	Muscle
Ceal_IPB996	*C. albifrons*	Barcelos, Amazonas	MPEG43856	Muscle
Ceal_IPB997	*C. albifrons*	Barcelos, Amazonas	MPEG43857	Muscle
Ceal_LB736	*C. albifrons*	Reserva de Desenvolvimento Sustentável Amanã	MPEG36634	Muscle
Ceal_LB772	*C. albifrons*	Reserva de Desenvolvimento Sustentável Amanã	MPEG36868	Muscle
Ceal_LB776	*C. albifrons*	Reserva de Desenvolvimento Sustentável Amanã	MPEG36869	Muscle
Ceal_LB858	*C. albifrons*	Parque Nacional da Amazônia	MPEG43778	Muscle
Ceal_LB1272	*C. albifrons*	RDS Cujubim	MPEG41791	Muscle
Ceal_LB1285	*C. albifrons*	RDS Cujubim	MPEG41802	Muscle
Ceal_LB1293	*C. albifrons*	RDS Cujubim	MPEG41801	Muscle
Ceal_LB1297	*C. albifrons*	RDS Cujubim	MPEG41800	Muscle
Ceka_KAAHOL	*C. kaapori*	Chega-tudo Municipality, Maranhão	MPEG43852	Muscle
Ceol_AP116	*C. olivaceus*	RDS do Rio Iratapuru, Rio Cupixi	MPEG43763	Muscle
Ceol_AP117	*C. olivaceus*	RDS do Rio Iratapuru, Rio Cupixi	MPEG43764	Muscle
Ceol_AP178	*C. olivaceus*	Parque Nacional Montanhas do Tumucumaque, Rio Mutum	MPEG43765	Muscle
Ceol_AP241	*C. olivaceus*	Parque Nacional Montanhas do Tumucumaque, Rio Anacui	MPEG43766	Muscle
Ceol_AP242	*C. olivaceus*	Parque Nacional Montanhas do Tumucumaque, Rio Anacui	MPEG43767	Muscle
SSR_1	*C. imitator*	SSR	N/A	Fecal
SSR_2	*C. imitator*	SSR	N/A	Fecal
SSR_3	*C. imitator*	SSR	N/A	Fecal
SSR_4	*C. imitator*	SSR	N/A	Fecal
SSR_5	*C. imitator*	SSR	N/A	Fecal
SSR_6	*C. imitator*	SSR	N/A	Fecal
SSR_7	*C. imitator*	SSR	N/A	Fecal
SSR_8	*C. imitator*	SSR	N/A	Fecal
SSR_9	*C. imitator*	SSR	N/A	Fecal

SSR_10	*C. imitator*	SSR	N/A	Fecal
SSR_11	*C. imitator*	SSR	N/A	Fecal
SSR_12	*C. imitator*	SSR	N/A	Fecal
SSR_13	*C. imitator*	SSR	N/A	Fecal
SSR_14	*C. imitator*	SSR	N/A	Fecal
SSR_15	*C. imitator*	SSR	N/A	Fecal
SSR_16	*C. imitator*	SSR	N/A	Fecal
SSR_17	*C. imitator*	SSR	N/A	Fecal
SSR_18	*C. imitator*	SSR	N/A	Fecal
SSR_19	*C. imitator*	SSR	N/A	Fecal
SSR_20	*C. imitator*	SSR	N/A	Fecal
SSR_21	*C. imitator*	SSR	N/A	Fecal
SSR_22	*C. imitator*	SSR	N/A	Fecal
SSR_23	*C. imitator*	SSR	N/A	Fecal
SSR_24	*C. imitator*	SSR	N/A	Fecal
SSR_25	*C. imitator*	SSR	N/A	Fecal
SSR_26	*C. imitator*	SSR	N/A	Fecal
SSR_27	*C. imitator*	SSR	N/A	Fecal
SSR_28	*C. imitator*	SSR	N/A	Fecal
SSR_29	*C. imitator*	SSR	N/A	Fecal
SSR_30	*C. imitator*	SSR	N/A	Fecal
SSR_31	*C. imitator*	SSR	N/A	Fecal
SSR_32	*C. imitator*	SSR	N/A	Fecal
SSR_33	*C. imitator*	SSR	N/A	Fecal
SSR_34	*C. imitator*	SSR	N/A	Fecal
SSR_35	*C. imitator*	SSR	N/A	Fecal
SSR_36	*C. imitator*	SSR	N/A	Fecal
SSR_37	*C. imitator*	SSR	N/A	Fecal
SSR_38	*C. imitator*	SSR	N/A	Fecal

SSR_39	*C. imitator*	SSR	N/A	Muscle
SSR_40	*C. imitator*	SSR	N/A	Fecal
SSR_41	*C. imitator*	SSR	N/A	Fecal
SSR_42	*C. imitator*	SSR	N/A	Fecal
SSR_43	*C. imitator*	SSR	N/A	Fecal
SSR_44	*C. imitator*	SSR	N/A	Fecal
SSR_45	*C. imitator*	SSR	N/A	Fecal
SSR_46	*C. imitator*	SSR	N/A	Fecal
SSR_47	*C. imitator*	SSR	N/A	Fecal
SSR_48	*C. imitator*	SSR	N/A	Fecal
SSR_49	*C. imitator*	SSR	N/A	Fecal
SSR_50	*C. imitator*	SSR	N/A	Fecal
SSR_51	*C. imitator*	SSR	N/A	Fecal
SSR_52	*C. imitator*	SSR	N/A	Fecal
SSR_53	*C. imitator*	SSR	N/A	Fecal
SSR_54	*C. imitator*	SSR	N/A	Fecal
SSR_55	*C. imitator*	SSR	N/A	Fecal
SSR_56	*C. imitator*	SSR	N/A	Fecal
SSR_57	*C. imitator*	SSR	N/A	Fecal
SSR_58	*C. imitator*	SSR	N/A	Fecal
SSR_59	*C. imitator*	SSR	N/A	Fecal
SSR_60	*C. imitator*	SSR	N/A	Fecal
SSR_61	*C. imitator*	SSR	N/A	Fecal
SSR_62	*C. imitator*	SSR	N/A	Fecal
SSR_63	*C. imitator*	SSR	N/A	Fecal
SSR_64	*C. imitator*	SSR	N/A	Fecal
SSR_65	*C. imitator*	SSR	N/A	Fecal
SSR_66	*C. imitator*	SSR	N/A	Fecal
SSR_67	*C. imitator*	SSR	N/A	Fecal

SSR_68	*C. imitator*	SSR	N/A	Fecal
SSR_69	*C. imitator*	SSR	N/A	Fecal
SSR_70	*C. imitator*	SSR	N/A	Fecal
SSR_71	*C. imitator*	SSR	N/A	Fecal
SSR_72	*C. imitator*	SSR	N/A	Fecal
SSR_73	*C. imitator*	SSR	N/A	Fecal
SSR_74	*C. imitator*	SSR	N/A	Fecal
SSR_75	*C. imitator*	SSR	N/A	Fecal
SSR_76	*C. imitator*	SSR	N/A	Fecal
SSR_77	*C. imitator*	SSR	N/A	Fecal
SSR_78	*C. imitator*	SSR	N/A	Fecal
SSR_79	*C. imitator*	SSR	N/A	Fecal
SSR_80	*C. imitator*	SSR	N/A	Fecal
SSR_81	*C. imitator*	SSR	N/A	Fecal
SSR_82	*C. imitator*	SSR	N/A	Fecal
SSR_83	*C. imitator*	SSR	N/A	Fecal
SSR_84	*C. imitator*	SSR	N/A	Fecal
SSR_85	*C. imitator*	SSR	N/A	Fecal
SSR_86	*C. imitator*	SSR	N/A	Fecal
SSR_87	*C. imitator*	SSR	N/A	Fecal
SSR_88	*C. imitator*	SSR	N/A	Fecal
SSR_89	*C. imitator*	SSR	N/A	Fecal
SSR_90	*C. imitator*	SSR	N/A	Fecal
SSR_91	*C. imitator*	SSR	N/A	Fecal
SSR_92	*C. imitator*	SSR	N/A	Fecal
SSR_93	*C. imitator*	SSR	N/A	Fecal
SSR_94	*C. imitator*	SSR	N/A	Fecal
SSR_95	*C. imitator*	SSR	N/A	Fecal
SSR_96	*C. imitator*	SSR	N/A	Fecal

SSR_97	*C. imitator*	SSR	N/A	Fecal
SSR_98	*C. imitator*	SSR	N/A	Fecal
SSR_99	*C. imitator*	SSR	N/A	Fecal
SSR_100	*C. imitator*	SSR	N/A	Fecal
SSR_101	*C. imitator*	SSR	N/A	Fecal
SSR_102	*C. imitator*	SSR	N/A	Fecal
SSR_103	*C. imitator*	SSR	N/A	Fecal
SSR_104	*C. imitator*	SSR	N/A	Fecal
SSR_105	*C. imitator*	SSR	N/A	Fecal
SSR_106	*C. imitator*	SSR	N/A	Fecal
SSR_107	*C. imitator*	SSR	N/A	Fecal
SSR_108	*C. imitator*	SSR	N/A	Fecal
SSR_109	*C. imitator*	SSR	N/A	Fecal
SSR_110	*C. imitator*	SSR	N/A	Fecal
SSR_111	*C. imitator*	SSR	N/A	Fecal
SSR_112	*C. imitator*	SSR	N/A	Fecal
SSR_113	*C. imitator*	SSR	N/A	Fecal
SSR_114	*C. imitator*	SSR	N/A	Fecal
SSR_115	*C. imitator*	SSR	N/A	Fecal
CC01	*C. imitator*	San Ramón	N/A	Blood
CC12	*C. imitator*	Manuel Antonio	N/A	Blood
CC13	*C. imitator*	Manuel Antonio	N/A	Blood
CC14	*C. imitator*	Manuel Antonio	N/A	Blood
CC15	*C. imitator*	Manuel Antonio	N/A	Blood
CC16	*C. imitator*	Manuel Antonio	N/A	Blood
CC17	*C. imitator*	Manuel Antonio	N/A	Blood
CC18	*C. imitator*	Manuel Antonio	N/A	Blood
CC19	*C. imitator*	Manuel Antonio	N/A	Blood
CC21	*C. imitator*	Manuel Antonio	N/A	Blood

CC23	*C. imitator*	Manuel Antonio	N/A	Blood
CC24	*C. imitator*	Manuel Antonio	N/A	Blood
CC25	*C. imitator*	Damas	N/A	Blood
CC26	*C. imitator*	Manuel Antonio	N/A	Blood
CC27	*C. imitator*	Manuel Antonio	N/A	Blood
CC28	*C. imitator*	Manuel Antonio	N/A	Blood
CC29	*C. imitator*	Manuel Antonio	N/A	Blood
CC30	*C. imitator*	Manuel Antonio	N/A	Blood
CC31	*C. imitator*	Manuel Antonio	N/A	Blood
CC32	*C. imitator*	Manuel Antonio	N/A	Blood
CC33	*C. imitator*	Liberia, Quebrada Grande	N/A	Blood
CC34	*C. imitator*	Liberia, Quebrada Grande	N/A	Blood
CC37	*C. imitator*	San Ramón	N/A	Blood
CC38	*C. imitator*	San Ramón	N/A	Blood
CC46	*C. imitator*	San Mateo	N/A	Blood
CC48	*C. imitator*	San Mateo	N/A	Blood
CC49	*C. imitator*	San Mateo	N/A	Blood
CC51	*C. imitator*	San Mateo	N/A	Blood
CC52	*C. imitator*	San Mateo	N/A	Blood
CC53	*C. imitator*	San Mateo	N/A	Blood
CC54	*C. imitator*	San Mateo	N/A	Blood
CC55	*C. imitator*	Palo Verde	N/A	Blood
CC56	*C. imitator*	Palo Verde	N/A	Blood
Saap_JA51	*Sapajus apella*	UHE Jatobá, Margem esquerda do Tapajós, Itaituba, PA	MPEG43822	Muscle
Saca_Cay500	*Sapajus apella (cay)*	Fronteira RO com MT - Cabixi/RO	MPEG43841	Muscle
Sali_DT02	*Sapajus apella (libidinosus)*	Delta do Parnaíba, Ilha Grande, Saquim, PI	MPEG43799	Muscle
Sama_CTG205	*Sapajus apella (macrocephalus)*	Rebio Abufari	MPEG43797	Muscle
Sani_1808	*Sapajus nigritus*	Porto Ferreira, Parque Estadual, SP	MPEG43816	Muscle
Saxa_DT07	*Sapajus xanthosternos*	Camacã, BA	MPEG43843	Muscle

Table S2. Summarization of sequencing results

Exon	No. individuals amplified	No. Miseq Reads	No. reads used post-QC (%)	Mean read pairs per individual	Min reads	Max reads
DRBe2	168	2,637,162	1,188,308 (45%)	4,243	129	40,239
DRBe3	167	1,991,254	1,207,198 (61%)	4,220	151	40,269
DQAe3	167	3,632,050	2,689,996 (74%)	9,406	64	86,839
DQBe3	164	3,807,242	1,204,764 (32%)	4,303	46	25,256

Table S3. Exact BLAST matches of allele sequences to *C. imitator* RefSeq genome assembly coordinates and transcript sequences

Exon	Query Sequence	Coordinates	Transcript ID
DRB, 2	Ceim-DRB*W:01	13746406 to 13746653	XM_017522743
	Ceim-DRB*W:02	13681151 to 13681398	XM_017522742
	Ceim-DRB*W:03	13834215 to 13834462	XM_017522746
			XM_017522745
			XM_017522744
DRB, 3	Ceim-DRB_15	13984209 to 13984422	
	Ceim-DRB_18	13749637 to 13749853	XM_017522743
			XM_017522742
	Ceim-DRB_19	13837632 to 13837848	XM_017522746
			XM_017522745
			XM_017522744
DQA, 3	Ceim-DQA_1	13454903 to 13455127	XM_017522741
	Ceim-DQA_2	13522420 to 13522644	XM_017522738
			XM_017522737
			XM_017522736
	Ceim-DQA_3	13624330 to 13624554	XM_017522739
DQB, 3	Ceim-DQB_1	13440805 to 13441058	XM_017522735
			XM_017522734
	Ceim-DQB_2	13509864 to 13510117	XM_017522446
	Ceim-DQB_3	13606851 to 13607104	XM_017522733
			XM_017522732
			XM_017522731
			XM_017522729

all coordinates come from scaffold accession NW_016107339.1

Table S4. Proportions of alleles that are unique to a single *Cebus imitator* population and those shared across two or more of the sampled populations in this study.

No. pops. with alleles	DRBe2	DRBe2 (%)	DRBe3	DRBe3 (%)	DQAe3	DQAe3 (%)	DQBe3	DQBe3 (%)
1	7	0.5	7	0.29	1	0.1	2	0.15
2	0	0	3	0.13	2	0.2	4	0.31
3	2	0.14	5	0.21	2	0.2	2	0.15
4	2	0.14	4	0.17	2	0.2	1	0.08
5	1	0.07	0	0	0	0	2	0.15
6	2	0.14	5	0.21	3	0.3	2	0.15
Total alleles	14		24		10		13	

Table S5. Number of shared alleles per exon across *Cebus* and *Sapajus* species.

	C. albifrons	*C. olivaceus*	*S. apella*	*S. nigritus*	*S. xanthosternos*
C. imitator	6/6/7/2	4/4/4/1	0/0/1/0	0/0/0/0	1/0/0/0
C. albifrons		7/6/5/1	0/1/0/0	0/1/0/0	1/0/0/0
C. olivaceus			1/2/1/0	1/1/0/0	0/0/0/0
S. apella				2/3/3/0	0/1/2/0
S. nigritus					0/0/1/1

*the cell format shows shared alleles for each locus as follows: DQB3/DQA3/DRB3/DRB2
